# QSpike tools: a generic framework for parallel batch preprocessing of extracellular neuronal signals recorded by substrate microelectrode arrays

**DOI:** 10.3389/fninf.2014.00026

**Published:** 2014-03-19

**Authors:** Mufti Mahmud, Rocco Pulizzi, Eleni Vasilaki, Michele Giugliano

**Affiliations:** ^1^Theoretical Neurobiology and Neuroengineering Lab, Department of Biomedical Sciences, University of AntwerpWilrijk, Belgium; ^2^Institute of Information Technology, Jahangirnagar UniversitySavar, Bangladesh; ^3^Department of Computer Science, University of SheffieldSheffield, UK; ^4^Brain Mind Institute, Swiss Federal Institute of Technology LausanneLausanne, Switzerland

**Keywords:** substrate arrays of microelectrodes, MEAs, extracellular, batch analysis, embarrassingly parallel signal-processing, cellular electrophysiology

## Abstract

Micro-Electrode Arrays (MEAs) have emerged as a mature technique to investigate brain (dys)functions *in vivo* and in *in vitro* animal models. Often referred to as “smart” Petri dishes, MEAs have demonstrated a great potential particularly for medium-throughput studies *in vitro*, both in academic and pharmaceutical industrial contexts. Enabling rapid comparison of ionic/pharmacological/genetic manipulations with control conditions, MEAs are employed to screen compounds by monitoring non-invasively the spontaneous and evoked neuronal electrical activity in longitudinal studies, with relatively inexpensive equipment. However, in order to acquire sufficient statistical significance, recordings last up to tens of minutes and generate large amount of raw data (e.g., 60 channels/MEA, 16 bits A/D conversion, 20 kHz sampling rate: approximately 8 GB/MEA,h uncompressed). Thus, when the experimental conditions to be tested are numerous, the availability of fast, standardized, and automated signal preprocessing becomes pivotal for any subsequent analysis and data archiving. To this aim, we developed an in-house cloud-computing system, named QSpike Tools, where CPU-intensive operations, required for preprocessing of each recorded channel (e.g., filtering, multi-unit activity detection, spike-sorting, etc.), are decomposed and batch-queued to a multi-core architecture or to a computers cluster. With the commercial availability of new and inexpensive high-density MEAs, we believe that disseminating QSpike Tools might facilitate its wide adoption and customization, and inspire the creation of community-supported cloud-computing facilities for MEAs users.

## Introduction

Among the most challenging open questions in Systems Neuroscience, structure-function relationship has raised a renewed interest. While novel ultrastructural anatomical investigations (Briggman and Denk, 2006; Mikula et al., 2012) promise to revolutionize the field, significant new progresses in our understanding of neuronal networks physiology and in preclinical neurotechnological applications, have been achieved by extracellularly monitoring the electrical activity of large neuronal ensembles (Rutten, 2002; Buzsaki, 2004; Schwartz, 2004; Wise et al., 2004; Lebedev and Nicolelis, 2006; Nicolelis and Lebedev, 2009). Complementary to high-resolution patch-clamp microscopic access and to mesoscopic non-invasive electroencephalography and functional magnetic resonance imaging, the extracellular interfacing of neurons to artificial devices has taken a considerable leap forward (Fromherz, 2006; Vassanelli et al., 2012; Spira and Hai, 2013).

Since its early introduction, extracellular recordings have been widely adopted both in academic and industrial pharmaceutical contexts, for monitoring and evoking neuronal activity *in vivo* and *ex vivo* under a variety of scientific, technological, neuroprosthetic, and clinical perspectives (Berdondini et al., 2006, 2009; Giugliano et al., 2008; Kim et al., 2009; Wang et al., 2012; Gortz et al., 2013; Liu et al., 2013). In addition, recent advances in real-time computing and in micro- and nanotechnologies opened brand new possibilities (Arsiero et al., 2007; Mazzatenta et al., 2007; Jain and Muthuswamy, 2008; Chen et al., 2009; Kim et al., 2009; Fendyur et al., 2011; Hai and Spira, 2012; Tian and Lieber, 2013).

However, in terms of data collection, analysis, and interpretation, multi-site extracellular recordings pose some challenges, given the large size of the raw data files acquired and the inherent complexity behind their rapid and accurate interpretation (Buzsaki, 2004; Stevenson and Kording, 2011). From Neuroinformatics perspectives, several open-source software toolboxes have been developed and released to the community over the years, addressing those issues and ultimately aimed at making electrophysiological data handling and analysis easier, faster, interactive, and more user friendly (Egert et al., 2002; Quiroga et al., 2004; Vato et al., 2004; Bonomini et al., 2005; Wagenaar et al., 2005; Morup et al., 2007; Cui et al., 2008; Huang et al., 2008; Magri et al., 2009; Novellino et al., 2009; Bologna et al., 2010; Abdoun et al., 2011; Kwon et al., 2012; Mahmud et al., 2012; Just et al., 2013). Hardware based techniques have been also made available to the community to perform spike detection and sorting (Yu et al., 2012; Hwang et al., 2013). Nonetheless, signal processing and data analysis remain intensive, even though modern personal computing power increased dramatically and costs steadily decreased. In such perspectives, the advantages of distributed data-analysis, cloud-based computing, computer clusters, and parallel graphical co-processing have become obvious to the neuroscience community (Wilson and Williams, 2009; Chen et al., 2011, 2013a,b), in analogy to what the field is witnessing for Computational Neuroscience applications.

**Figure 1:**
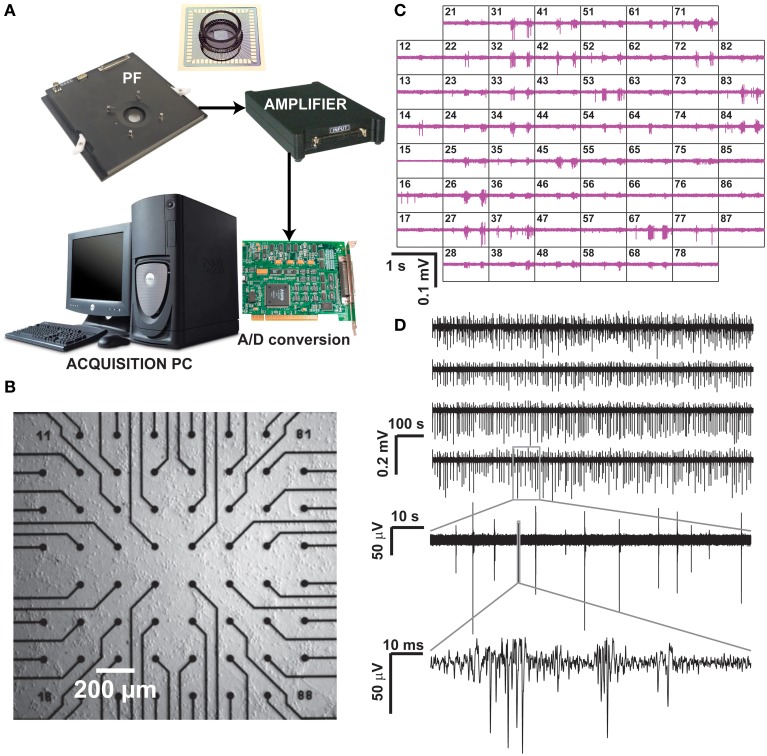
**Recording of neuronal activity *ex vivo*, by means of a commercial MEA hardware platform**. Our experimental setup **(A)** is composed by a pre-amplification and filtering (PF) stage and by an additional signal amplifier, connected via an A/D board to a dedicated data acquisition PC, which also controls a temperature controller and electrical stimulus generation (not shown). After plating and culturing mammalian primary cortical neurons *ex vivo* on a MEA for several days, spontaneous electrical activity is detected and recorded at each microelectrode **(B)**, arranged as a regular 8 by 8 layout, 200 μm spacing, and displayed in real time **(C)**. Representative raw voltage traces from four sample microelectrodes, recorded over 20 min, are sown in **(D)** with increasing levels of magnification, to reveal the stereotypical pattern of spontaneous multi-unit electrical activity.

In this work, we addressed some of the basic requirements of substrate-integrated microelectrode arrays (MEAs) users, focusing on routine multichannel data analysis in *in vitro* studies, where several experimental conditions are examined and several binary raw data files are collected daily. We defined two major objectives that we consider a priority in our own laboratory: (i) increasing experimental throughput by freeing the data-acquisition computers from the burden of subsequent raw-signal analysis; (ii) providing the end-user with software tools that could be employed with neither previous training nor computer proficiency, but still easily customizable to include any analysis algorithm. To this aim, we developed and implemented a web-based workflow, named QSpike Tools, for the unsupervised execution of generic signal preprocessing and analysis of multichannel extracellular signals (see Figure 1). As sample data processing primitives for demonstration purposes, we chose a minimal set of basic operations that are performed in any multi-unit activity analysis: filtering, peak-detection, sorting, and simple spike-rate analysis. Tedious and long interactive analysis sessions could be then replaced by an automated procedure, and most important for us, by a more rational and efficient use of the existing shared computing resources in our laboratory. This was accomplished by delegating and batch queuing the preprocessing of the raw data files to an in-house multi-core server. This is controlled and monitored remotely via a simple web browser, with no (computing) programming familiarity required, and leaving part of the resources of the server free for other users. Our generic framework might be successfully applied, or easily customized to include additional analysis scripts (e.g., in MATLAB), in the context of routine compounds screening, with highly consolidated analysis methods and with a set of established performance indicators. We also ultimately aimed at a scenario where no further manipulation of post-processed data may be required to the end-user, with one of the outcomes of the automated analysis being a portable document format (PDF) report, containing textual information as well as automatically generated tables, graph, and plots (see the Supplementary Material).

We are convinced by the importance of disseminating QSpike Tools to the community, as a generic, easily customizable, processing workflow, for the sake of its potential wide adoption. Indeed, robust open-source distributed (grid) platforms are often in use in many laboratories or (super)computing departmental facilities. Finally, inspired by the recent creation of community-supported Neuroinformatics shared facilities for numerical simulations, such as the NSF-funded Neuroscience Gateway Portal (NSG^1^), our work could lead to the creation of institutional or international facilities for remote automated MEA data analysis.

## Materials and methods

### Multi-electrode array recordings of neuronal multiunit activity

Commercial microelectrode arrays (MEAs) for *in vitro* electrophysiology were obtained from Multichannel Systems (Reutlingen, Germany) and employed in routine experiments (Figure 1). Briefly, MEAs consists of 60 Indium Tin Oxide (ITO) planar microelectrodes (30 μm in diameter, 200 μm in spacing) with 8 by 8 regular layout, microfabricated on a glass substrate by photolithography, reactive ion etching, and physical vapor deposition. Prior to cell seeding, MEAs were autoclaved and coated by Polyethyleneimine (0.1% PEI, Sigma-Aldrich). Primary cortical cell cultures were obtained by standard methods from newborn Wistar rats (Charles River, France), following national and institutional guidelines on animal experimentation, upon enzymatic (0.025% trypsin) and mechanical dissociation. Prior to seeding, cells were centrifuged and suspended in a medium containing Modified Essential Medium supplemented with 2 M glucose, 200 mM l-glutamine, 50 μg/mL gentamycin and 5% horse serum (Sigma-Aldrich). Approximately 2000–3000 cells/mm^2^ were plated on the inner area of each MEAs and maintained in culture medium (changed three times per week), at 100% relative humidity, 37°C and 5% CO_2_ for 1–30 days *in vitro* (DIV). MEAs were sealed by fluorinated Teflon membranes, allowing gas but no water exchanges, reducing osmolarity alterations and contamination risks (Potter and DeMarse, 2001), making possible to perform the recordings in a low-humidity, electronic-friendly, conditions at 37°C, 5% CO_2._

The MEA microelectrodes were then employed to monitor non-invasively the collective electrical activity of neuronal networks developing *ex vivo* on their substrates. Recordings took place after 21 DIV upon mounting of each MEA into the recording amplifier (Figure 1A, 1060BC, Multichannel Systems, Reutlingen, Germany) and acquiring 15–30 min of spontaneous electrical activity was acquired at 25 kHz/channel, after 1200x amplification. MC Rack software (Multichannel Systems, Reutlingen, Germany) was employed to store the digitized data on disk, as multiplexed binary files (^*^.mcd file format), with each file containing raw voltage waveforms from all the MEA microelectrodes.

Additional hardware (Figure 1A) included an acquisition computer with a PCI analog to digital board (MC Card, 64 channels A/D, 4 DIO, 16bits Multichannel Systems, Reutlingen, Germany), as well as a temperature regulator and a stimulus isolator (STG1002, Multichannel Systems, Reutlingen, Germany). Figure 1B depicts a magnification of the inner area of a MEA (i.e., 1 × 1 mm^2^) populated by microelectrodes, and Figure 1C displays a typical recording session where the extracellular electrical activity sensed at each microelectrode can be monitored over time as an electrical potential. Figure 1D reports representative raw (off-line band-pass filtered) sample recordings, acquired over 20 min from six sample microelectrodes.

### System architecture

**Figure 2:**
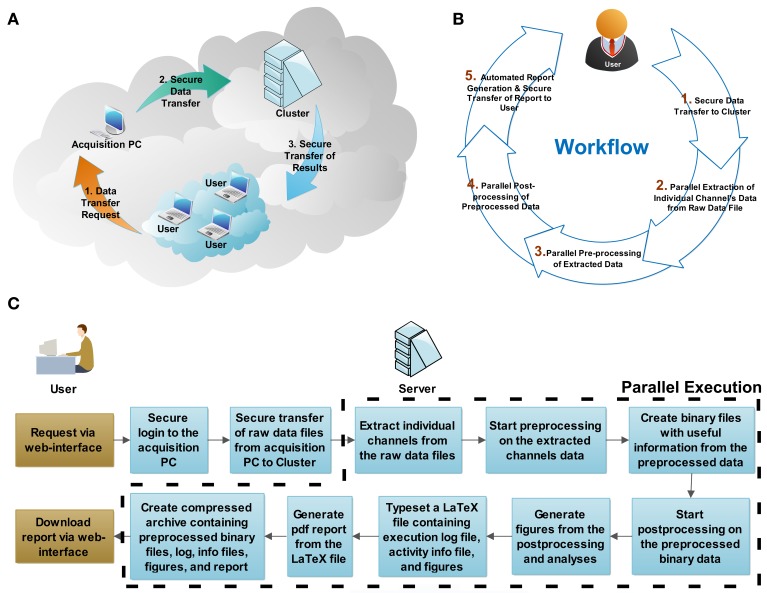
**System architecture and workflow description**. The physical configuration of computers within network(s) is reminiscent **(A)** of a cloud-computing architecture. Through a web browser, the user initiates **(B)** the workflow by requesting secure data transfer from the acquisition computer to the server, and by starting the parallel batch processing and analysis. At the end of the analysis, the server generates automatically a portable document format (PDF) document, reporting on the execution log, the activity summary, and the result summary, which is incorporated in a compressed file archive along with the analysis result. When ready, the user concludes the session by securely downloading the compressed file archive to his/her personal computer. The flowchart **(C)** shows the actions required by the user and the processing steps executed by the server (cluster). The steps executed in parallel are outlined by the dashed line.

The QSpike Tools workflow is based on a client-server architecture (Figure 2A). The server is a stand-alone (powerful) computer workstation, or it is the master-node of a computers cluster, running a standard distribution of the Linux operating system. Accordingly, the individual processor cores of the server, or the computers of the cluster are configured as distributed computing nodes, as in a high-performance computing intranet. The master node also runs a web server software, capable of launching a series of server-side operations (e.g., via the common gateway interface, CGI^2^, as in web applications) when instructed by a client computer connected to the same intranet.

**Figure 3:**
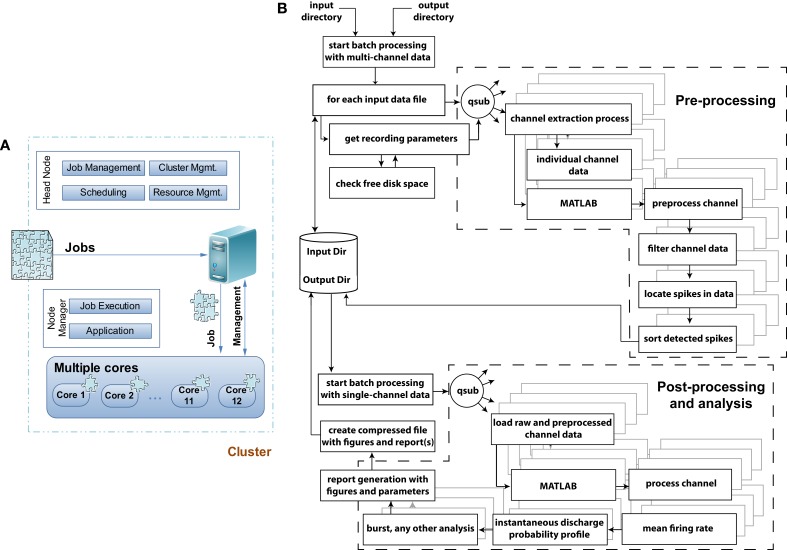
**Grid computing architecture and flowchart of the data pre-, post-processing, and analysis module. (A)** When jobs arrive at a grid computing environment, the jobs are decomposed into individual jobs and allocated to each processor core or cluster for completion. In a multicomputer cluster system the head node of the cluster is responsible for job-, cluster-, resource-management and scheduling leaving the application and job execution on the individual nodes of the cluster system. In QSpike Tools the benefit of a multicore single computer cluster has been exploited where the responsibilities of the head and individual nodes are handled by the grid computing software. **(B)** Flowchart of the execution model with exemplary pre-, post-processing and analysis modules to demonstrate and distinguish among the operations in terms of their parallelization. Dashed sections outlines the two major sets of operations, where parallelization of the tasks takes place. The remaining tasks instead operate serially. The parallelization is achieved by the *qsub* command, provided by the grid-scheduling environment, distributing the batch-queued jobs to the specified cores for their parallel execution.

The preprocessing performed by QSpike Tools includes five steps (Figure 2B). Some of them occur and progress automatically in a sequence, while others are only initiated via user interaction with the web page hosted by the master node upon selection appropriate hyperlinks. The links provide:

1. A fast and secure SFTP^3^ raw data transfer, from the data acquisition setup storage hard drive to the computer(s) dedicated for data preprocessing;
2. The conversion of each multiplexed raw data file into several binary files, each containing data points from a distinct recording channel;
3. The scripting-based (i.e., written in MATLAB, The Mathworks, Natick, USA), fully extensible, preprocessing sequence for each file, currently including band-pass filtering, stimulation-artifacts removal, multi-unit activity detection and elementary spike-sorting (Figure 3B);
4. The additional scripting-based (i.e., MATLAB) visualization of the (multi)unit activity extracted by the previous steps (e.g., MEA-wide synchronous bursting rate, single-channels and MEA-wide firing rate, intra-burst instantaneous discharge probability, etc.);
5. The automated typesetting of a PDF report from a dynamically generated and compiled LaTeX^4^ source file, including both textual and graphical information, extracted by the previous step and secure download of the PDF report and of all intermediate and final preprocessed files (e.g., spike time-stamps, spike waveforms, spike count) to the user's personal computer, as a compressed file archive.

Provided that certain dependencies are respected (e.g., step 4 must follow the completion of step 3, across all electrodes of the same data file), all the remaining steps (i.e., 2–5) can also be batch-queued and executed in parallel (e.g., one task per core, processor, or intranet node). A flowchart explaining the detailed processing steps is shown in Figure 2C.

The QSpike Tools workflow is in fact based on the observation that any CPU-intensive preprocessing needed can be executed in parallel, independently of any other recorded channel (Denker et al., 2010). All operations, necessary for any subsequent data analysis, can be performed in parallel across channels thus directly exploiting the advantages of embarrassingly parallel scheduling (Figure 3).

It is worth to note that, in our context, parallel processing implies performing pre-, post-processing and analyses on each recorded channel, independently. This excludes sophisticated spike detection, sorting, and analyses algorithms that are useful when employing, e.g., tetrode-like arranged MEAs, whose inter-electrode distances are much smaller than those employed here. In those circumstances, correlated information from distinct channels cannot be treated independently and must be jointly analyzed. While the series of analyses of QSpike Tools can be extended to include similar analysis algorithms, this has not been yet implemented in its current version as it implies a redesign of its principle of operation, beyond the embarrassingly parallel computing.

### System implementation

The system has been tested on a multi-core personal workstation (Precision, T7500, Dell, Asse-Zellik, Belgium), equipped with two six-core Xeon processors and 24 GBytes of shared memory, running the Ubuntu^5^ 10.10 server operating system, the Apache^6^ webserver software, and MATLAB R2012a. In addition, a basic grid-computing environment was installed and set up, using the (now outdated) Sun Grid Engine (SGE, Sun Microsystems, Santa Clara, California), or the equivalent Open Grid Scheduler/Grid Engine^7^. The last implements a scheduling system for the management of distributed computing resources (i.e., individual cores, processors, and computers) and it enables the definition of one or more computing queues. Upon launching a job by a special command of the scheduler, while assigning it to a specific queue, the operating system is not anymore in charge of balancing the computing load on the entire computer architecture. Instead, that job is scheduled for execution and assigned to an individual unused node, among those reserved. As mentioned, these nodes may be the processor cores of the server, as in our case, or—transparently for the user—the cores of the processor(s) of Ethernet-connected computers (Figure 2A).

Both the client PC(s) and the workstation run the OpenSSH server software, which provides a secure file transfer protocol (SFTP)^8^. With the typical size of a MEA raw data file (e.g., 60 channels/MEA, 16 bits A/D conversion, 20 kHz sampling rate: approximately 8 GB/MEA,h uncompressed), we found that scripted command-line SFTP performed better than drag-and-drop over network mounted shares or graphical user interface clients. Via scripted SFTP and by employing a gigabit Ethernet switch (Catalyst 2960S-48TS-L, Cisco, Diegem, Belgium), the MEA data transfer rate was consistently approximately 100 MBps, in our daily tests.

Figure 3A sketches the simple structure of the grid-computing environment, and of the way we benefit from it. Upon arrival of a preprocessing job request, the large multiplexed (^*^.mcd) binary file is decomposed into 60 individual files, containing the voltage raw waveforms of each microelectrode. Then, the same operations (Figure 3B) are applied and repeated identically to each files, so that the original job is distributed in parallel to the allocated cores. The server performs the management task of submitting the jobs to the execution queue and of checking for a free node (60 channels over, e.g., 12 cores implies an overall load of 5 jobs/core), in a way fully transparent to the end-user. The node manager is ultimately responsible for the execution of each parallelized task, and logs (standard and error) output diagnostics as separate text files.

To favor readability and user customization, most of the data transfers, user communication, job decomposition, and scheduling were coded as Bash shell^9^ scripts. Signals preprocessing, analysis, and automated generation of figure plots were implemented as MATLAB scripts.

The flow chart of Figure 3B depicts the various components of the preprocessing and analyses pipelines, and indicates (i.e., by dashed line boxes) the execution streams that operate in parallel. As mentioned in the Introduction, the user initiates the execution of a series of sequential steps, where input and output folder names are first provided to the non-interactive Bash scripts, which fetch the (list of) input raw data file(s) and store output results (e.g., the PDF report, the time-stamp of peak-detected multiunit activity, the analog waveform of each detected event for subsequent offline spike-sorting). Then, the individual channels and their preprocessing are distributed simultaneously as independent jobs among the cores using the qsub command, making the analysis trivially parallelized and limited only by the number of nodes available to the queue.

The decomposition of the raw data into the individual channels is the first step in the preprocessing pipeline: it is performed by a custom code, written in C and based on the vendor data access API.

After the extraction of individual channels, further preprocessing like a causal signal filtering (Quian Quiroga, 2009), robust peak-detection detection, elementary spike sorting (i.e., as positive or negative threshold crossings), and spike waveform storage are performed (Quian Quiroga, 2012). For instance, filtering is based on a band-pass, zero-phase digital filter of fourth order (i.e., by filtfilt^10^ and ellip^11^ MATLAB functions, included in its Signal Processing Toolbox^12^) between 400 and 3000 Hz, while peak detection is based on the evaluation of the median of the raw trace, following the sample code of Wave_clus^13^ (Quiroga et al., 2004), which was chosen as our golden standard. The final result of the peak-detection is the conversion of the analog raw voltage waveforms into a time-series, for each channel: these are the time-stamps of the multiunit activity, which are stored for further analyses as simple text files. As soon as these are available for all channels, they are consolidated in a single file and ready for further MATLAB-based analysis.

The post-processing and analyses pipeline starts with loading the raw single channel data, made available by the channel extraction process, and the files saved during the preprocessing stage. Analyses such as spike-train feature extraction, firing rate estimation, the instantaneous discharge probability profile calculation (Van Pelt et al., 2004), and network-wide synchronized bursting frequency estimation (Bologna et al., 2010) are performed on the preprocessed data. Each of them produces one or more figures, as well as numerical information that are also included in the portable document format (PDF) report generated—see the Supplementary Material. Finally, the report, the figures, and all the intermediate (text and MATLAB binary) files are compressed as a file archive: for each raw data file provided initially, a single file archive is generated by the workflow and available for subsequent user's download.

## Results

### Performances and sample preprocessing

**Figure 4:**
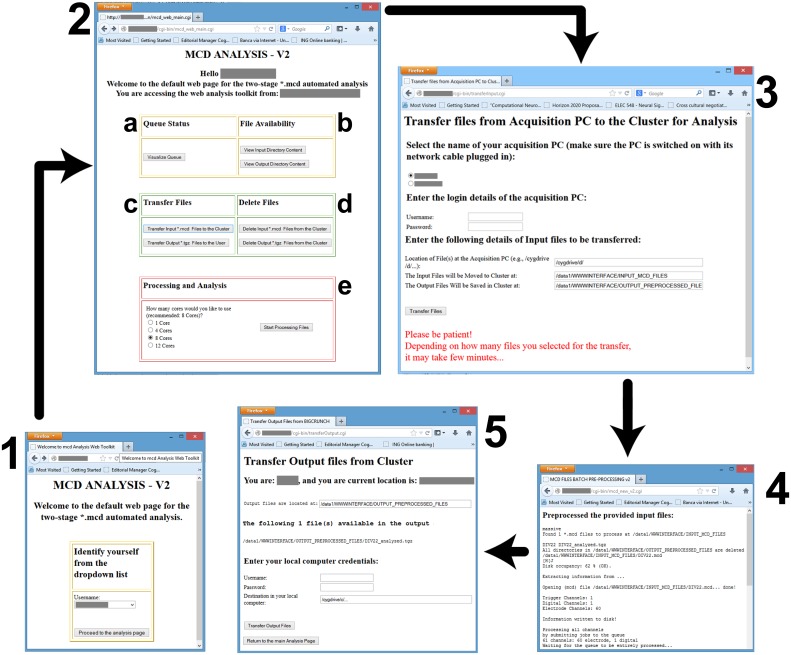
**Screenshots of the web-interface**. Each window is numbered to denote a separate stage of the workflow, and consist in: **(1)** the user-identification; the **(2)** main control webpage; the **(3)** file transfer interface from the data acquisition PC to the master node; **(4)** the result of the preprocessing; and **(5)** the file transfer from the master node to the user PC. Individual letter labels in **(2)** represent grouped functionalities, such as the visualization **(a)** of the status of the computing queues, the availability check **(b)** for the data files in the input and output directories, the file transfer and management functions **(c,d)**, and finally **(e)** the initiation of the parallelized preprocessing and analysis, with an option to select the destination queue.

The workflow discussed in the previous sections, has been employed daily in our laboratory and extensively tested. For demonstration purposes, we selected typical data files, containing the spontaneous electrical activity of *ex vivo* developing networks of primary cortical neurons, and we provide the PDF report automatically generated by QSpike Tools of its analysis as a Supplementary Information. By a simple web interface, as shown in Figure 4, where the various Bash scripts are linked, we experienced that several users with limited computer proficiency could perform smoothly server-side operations, such as visualizing the status of the server queues, clearing the input and output directories from previous data analysis sessions, initiating file transfer, and finally launching the data preprocessing. The same operations could be also performed remotely, form home, upon the virtual private network (VPN^14^) imposed by our university.

Figure 4 shows a screenshots of the web-interface of QSpike Tools. Upon navigating to the web address of the server, the user is presented with page 1, where an identification is required. This takes the user to the page 2, containing hyperlinks to most of QSpike Tools functionalities: (a) visualizing the current status of the queue, (b) checking the correct availability of the (transferred) files in the input/output directories, (c) transferring data and report to and from the server, (d) clearing the input/output directories of their content upon completion of the analysis, and (e) starting the work-flow by selecting the required number of cores to be used. Once the user opts to transfer raw data files to the server, page 3 appears with additional options. At the end, page 4 is shown with progress and diagnostic information. Finally, as the user wants to download the report, page 5 appears and enables downloading the corresponding file to the user's PC.

For the sake of testing and comparison, we configured and run QSpike Tools on sample binary (^*^.mcd) data files of two different sizes (i.e., approximately 1.5 GB for 8 min and approximately 3.5 GB for 20 min recording). As the execution times depend on both the raw data file and the number of multiunit events, for the sake of fair comparison we executed repeatedly QSpike Tool analysis for 10 times over the very same files. We specifically selected two files, from each file size groups.

**Figure 5:**
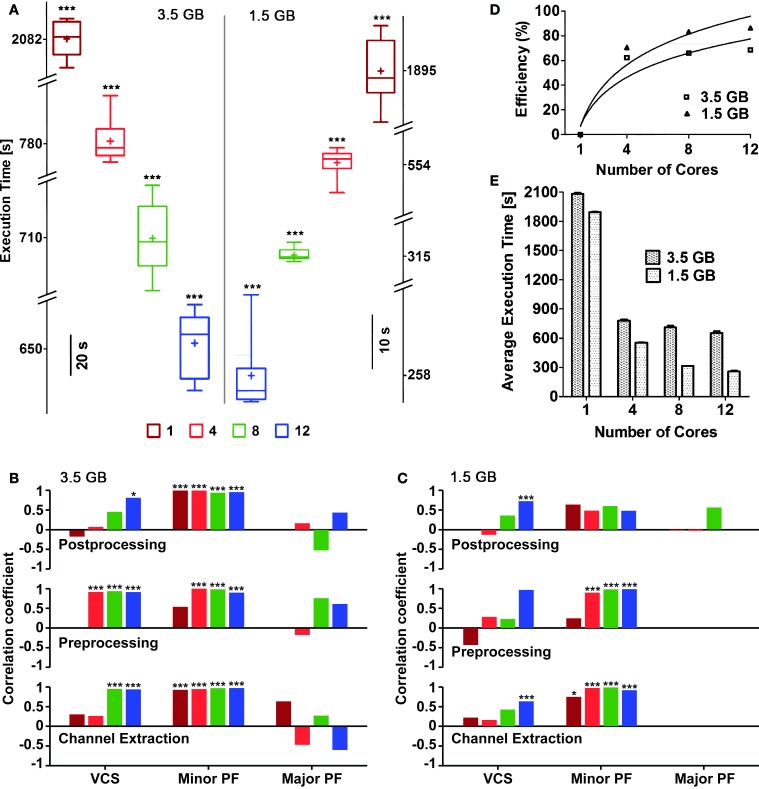
**Execution times and efficiency, for an increasing numbers of cores reserved**. Box plots **(A)** with box height showing 25–75% of the sample values were used to represent maximum and minimum (whiskers), median (“−”) and mean (“+”) execution times, respectively, which were all significantly different (*p* < 0.05) based on the number of cores used. The vertical line in the middle separates the data representation corresponding to distinct file sizes (3.5 vs. 1.5 GB). Pearson's liner correlation coefficients **(B,C)** show that the execution times of individual sub-processes are correlated to, certain computationally intensive operations performed by the operating system such as, minor page faults (Minor PF), and voluntary context-switching (VCS) (^***^p < 0.0001; ^*^p < 0.05) in case of large file size **(B)**. Though major page faults (Major PF) were noticed while analyzing the large file, they had either negative or no correlation to the execution times. The execution times of the first two sub-processes for the small file were mainly correlated to Minor PF (^***^p < 0.0001; ^*^p < 0.01) **(C)**. Overall, negligible amount of Major PF occurred during the execution of the small file and only when large number of cores was used, correlation between execution times and VCS were noticed. The bars in **(B,C)** are plotted using the same color code of **(A)**. The efficiency of parallelization was also quantified **(D)** as referred to the slowest execution time when a single core was used: continuous lines are best-fit logarithmic plots, whose mean squares were 0.8807 and 0.9306 for 3.5 and 1.5 GB file sizes, respectively. The mean execution times **(E)** for both file sizes also show the reduction of the execution time, for an increasing number of cores available.

We employed four distinct predefined queues (i.e., with 1, 4, 8, and 12 reserved cores) to compare the User Execution Times^15^ (Figure 5). Confirming the embarrassingly parallelization of the task, we found that execution time reduced significantly (*p* < 0.05, ANOVA; sublinearly) with an increasing number of cores available (see Figure 5A), with maximum and minimum execution times ranging from 34.7 min ± 10 s to 10.8 mins ± 17 s or from 31.5 min ± 5 s to 4.3 min ± 6 s for large and small files, respectively.

Despite input files were identical, their repeated analysis led to variable execution times. In order to trace the sources of such variability and provide a preliminary profiler analysis of QSpike Tools, we considered separately the steps performed during the entire workflow, launching manually three subprocesses: (i) raw data channel demultiplexing, (ii) pre-processing of analog voltages (Figure 3B), and (iii) post-processing (Figure 3B). We then monitored, by standard Linux system calls, the occurrence of three computationally intensive operations managed by the operating systems: voluntary context-switching^16^ (VCS), minor page faults^17^ (Minor PF), and major page faults (Major PF).

We found that the execution times are significantly correlated to the occurrence of Minor PF and of VCS, in case of the large input file (Figure 5B) (*p* < 0.0001 and *p* < 0.05, respectively). The same occurs for smaller input files, particularly during channel extraction and pre-processing sub-processes (Figure 5C).

We then noticed that the execution time with the highest number of cores was found to be more sensitive to page faults and context-switching. This may be explained in terms of the fixed amount of physical memory, as its allocation per core decreases with increasing number of cores. As a consequence, memory demanding jobs required to run in parallel will have less allotted memory and result in frequent page faults and context-switches (Tay and Zou, 2006). Based on the necessity one may try to reduce the occurrence of page faults by implementing available methods in the literature for better memory management (Zhou et al., 2004).

The efficiency E across distinct queue size, was calculated with respect to the execution times required by a single-core system as *E* = *T_max_*/Δ*T* × 100%, with *T_max_* the execution time of the slowest execution—referred to a single core. As expected, the execution efficiency increases (Figure 5D) and the mean execution time maintains a decaying trend (Figure 5E) with increasing number of cores used.

We have excluded those steps when computing the execution times, such as file transfer to and from the master node file system, since these are dependent on physical characteristics of the Ethernet network as well as of user interactions. The significant differences in the execution times indicate that using more powerful computers with significantly large number of cores is advantageous for a large set of raw data files. We note however that suboptimal memory management by MATLAB parallel instances still deserve attention, as the proportional decrease in the average execution time for large data files differed from the execution time for smaller files.

**Figure 6:**
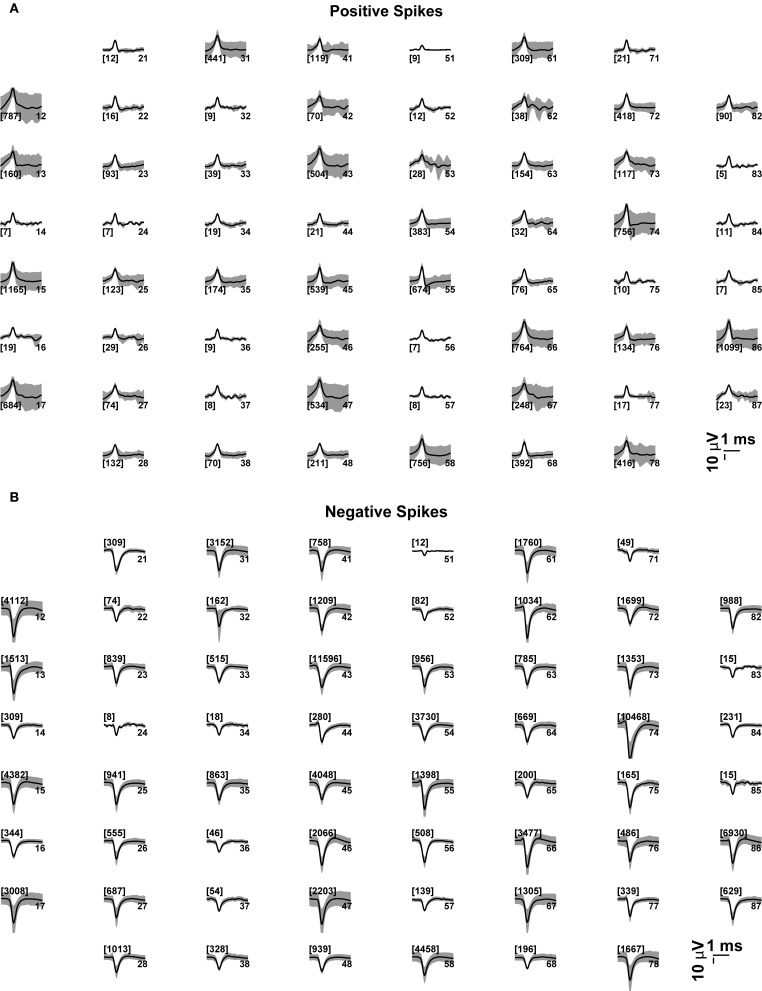
**Average voltage waveforms, corresponding to (positive or negative) peak-detection events at each microelectrode**. Each subplot represents graphically the 8 × 8 layout of the MEAs employed in this study (see the Methods). The average voltage trajectories (thick black lines) of the positive **(A)** or negative **(B)** threshold crossing events are displayed for each microelectrode, together with its point by point variability (gray shading, i.e., standard deviation), and with the number of events among brackets. The numbers on the right hand side of each subplot denote the labeled electrode name.

For the sake of illustration, we further comment and discuss briefly some of the standard analyses performed by QSpike Tools. The first step in the analysis pipeline is to display graphically the waveforms detected at each electrode by the peak-detection algorithm. This allows an elementary spike sorting procedure, discriminating between positive- or negative-threshold crossing. It also enables the user to perform a quantification of the average voltage data trajectory amplitude, its confidence interval, as well as the overall number of events detected. Besides serving as a quality assessment of the raw signals recorded and of the viability of the culture examined (Figure 6), next to each subplot the indication of the total number of multiunit events detected at every electrode provides immediate feedback on the significance of the average waveform displayed therein. This is also particularly useful when distinct microelectrode materials are used (e.g., carbon nanotubes or nano-crystalline diamonds coated electrodes), and when the majority of events detected by a given electrodes are monophasic, biphasic, or triphasic extracellular action potentials.

**Figure 7:**
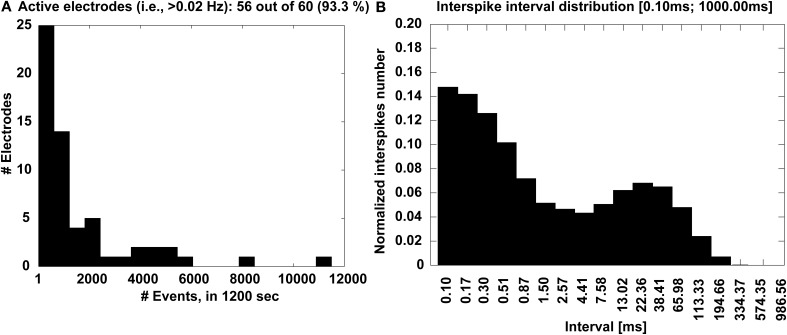
**Experimental outcome at a glance**. The distribution of the number of electrodes **(A)** that detected activity with significant occurrence frequency is displayed, together with the MEA-wide distribution **(B)** of the interspike intervals that reveal a bimodal profile, corresponding to the recurring transient, MEA-wide synchronization of the electrical activity.

Complementary information is also displayed in the form of a histogram (Figure 7A), which quantifies the number of microelectrodes that detected a sufficiently large number of events, higher than a 0.02 Hz minimal occurrence frequency. Finally, the interspike interval (ISI) distribution is also provided across the entire MEAs, merging together all the events (Figure 7B), to ultimately reveal whether or not a bimodal distribution is present and corresponds to a mature bursting electrical phenotype of the culture.

**Figure 8:**
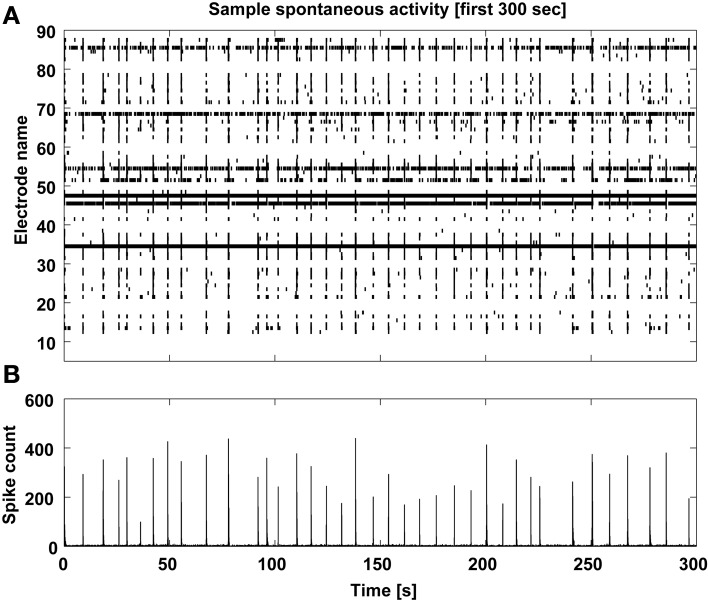
**Sample spontaneous activity display**. The multiunit spontaneous activity is displayed as raster-plot **(A)** across the detecting microelectrodes for the first 5 min of each data file, and its corresponding spike count is computed **(B)** to reveal the MEA-wise stereotypical episodic synchronization of neuronal activity.

**Figure 9:**
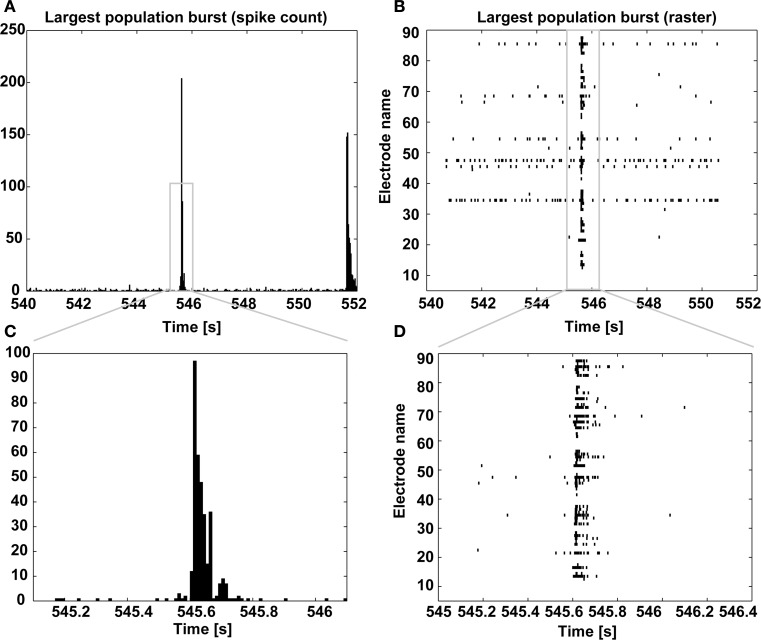
**Largest population burst**. As in Figure 8, the spike count is displayed and it is centered on the largest MEA-wide synchronization event (i.e., a burst) **(A)**, together with its corresponding spike raster diagram **(B)**, with increasing temporal resolution **(C,D)**.

Finally, a graphical display of the first few minutes of each recording is also produced, as a raster plot of the individual spike times across channels (Figure 8). In our own experience, this provides a rather immediate overview of the recording session and on the viability of the culture. Based on a simple eyeball estimation of the occurrence of MEA-wide synchronized events, and from a detail of the largest synchronized event at increasing temporal resolutions (Figure 9), a non-expert user can gain immediate insight on whether the typical expected electrical patterned activity occurred and the extent of the most notable synchronous transient, useful when very small recordings are performed.

The pdf-report, generated automatically at the end of the preprocessing, is provided as Supplementary Information, and offers diagnostic details on the execution of the workflow (i.e., the queue name, start and end time of the processes, and total execution time), as well as of the quantitative information about the data (i.e., total recording duration, total number of samples, sampling rate, number of active electrodes, number of spikes, number of bursts, mean burst duration, standard deviation of burst duration, mean and standard deviation of the inter-burst-intervals, burst detection threshold, etc.).

## Discussion and conclusion

Starting in the early 2000s, the MEA-Tools (Egert et al., 2002) laid the foundation for user-friendly analysis of MEA data, and eventually became a platform-independent, open source framework for the analysis of neuronal activity data, called FIND (Meier et al., 2008). The toolbox provided for the first time the community with a convenient graphical user interface, and with a set of MATLAB routines, for accessing, visualizing, and analyzing MEAs data. Its minor shortcoming of being centered to a specific hardware system was finally overcome by the DATA-MEAns (Bonomini et al., 2005), which operates and produces ASCII files to be used by other graphical, mathematical or statistical packages. The Neural Signal Manager package (Novellino et al., 2009) was then designed to perform sophisticated analysis of spike and bursting activities, and the SPYCODE package (Bologna et al., 2010) increased the repertoire of standard and advanced tools including cross-correlation analysis and neuronal avalanche detection. MEABench (Wagenaar et al., 2005), on the other hand, was designed to provide advanced means at real-time control of the data acquisition hardware, removal of stimulation artifact, detection of spikes, visualization of voltage traces with spike raster plots.

Instead of investing on our own featured package or toolbox, we explicitly focused on the most time-consuming aspect of preprocessing large MEA data files. We do understand that MATLAB is an interpreted language and is not the fastest possible solution to perform stereotyped operations (e.g., filtering and peak-detection). Nonetheless, it has inherent advantages that we strongly favored it as many (new) algorithms are very often proposed, shared, and validated by the community as MATLAB code, making their rapid prototyping or implementation easier. We aimed at taking advantage of those existing analysis tools, but only handling much smaller and portable preprocessed files and we obtained a significant overall reduction in the analysis. For some applications (e.g., a pharma industrial context), however a minimal set of basic analyses and their automated reporting as a PDF file, as we demonstrated here, could instead be sufficient to increase user access and throughput of MEA analysis.

QSpike Tools should be then considered as complementary to existing tools, and its advantages make it suitable for performing automated preprocessing of large datasets, prior to any user interactive (advanced) analysis session.

The limitation of the current version of QSpike Tools is the compatibility with a single proprietary input file format (i.e., ^*^.mcd), as generated by the acquisition software of the commercial platform we use (i.e., Multichannel Systems). Overcoming this limitation is a task for the future and it will be rather simple, in all the cases of raw data formats for which a file interpreter is already available for MATLAB, under Linux. Along these lines, we will also rely on community-supported vendor-neutral initiatives, such as the Neuroshare API library of functions (http://neuroshare.sourceforge.net). In addition, we also aim at (i) enriching the user interface of QSpike Tools, (ii) including a user-specific configuration file to enable custom sets of analyses, and (iii) extending QSpike Tools to *in vivo* data.

Along the lines of the creation of community-supported Neuroinformatics shared facilities for numerical simulations, we launch the proposal for one or more facilities dedicated to automated MEA data analysis. Finally, our work will be made available through the International Neuroinformatics Coordinating Facility, INCF (http://incf.org/) website as well as at https://sites.google.com/site/qspiketool for the community.

## Information sharing statement

QSpike Tools and its installation manual are available on request from https://sites.google.com/site/qspiketool.

## Authors and contributors

This work was carried out in close collaboration between all co-authors. Eleni Vasilaki and Michele Giugliano first defined the research theme and contributed an early software architecture. Mufti Mahmud, Rocco Pulizzi, and Michele Giugliano further implemented and refined methods and algorithms, carried out the data analysis, and preprocessing routines design. Mufti Mahmud and Michele Giugliano wrote the paper. All authors have contributed to, seen and approved the final manuscript.

### Conflict of interest statement

The authors declare that the research was conducted in the absence of any commercial or financial relationships that could be construed as a potential conflict of interest.

## Footnotes

^1^http://www.nsgportal.org/

^2^http://en.wikipedia.org/wiki/Common_Gateway_Interface

^3^http://www.openssh.com

^4^http://www.latex-project.org

^5^http://www.ubuntu.com/download/server

^6^http://httpd.apache.org

^7^http://gridscheduler.sourceforge.net

^8^**(Win)** http://www.cygwin.com, **(OS X)** http://www.maclife.com/article/howtos/how_enable_ssh_your_mac

^9^http://en.wikipedia.org/wiki/Bash_(Unix_shell)

^10^http://www.mathworks.com/help/signal/ref/filtfilt.html

^11^http://www.mathworks.com/help/signal/ref/ellip.html

^12^http://www.mathworks.nl/products/signal/

^13^http://www2.le.ac.uk/centres/csn/wave-clus-docs/

^14^http://en.wikipedia.org/wiki/Virtual_private_network

^15^http://en.wikipedia.org/wiki/Time(Unix)

^16^http://en.wikipedia.org/wiki/Context_switch

^17^http://en.wikipedia.org/wiki/Page_fault
