# Supplementary material for: QSpike tools: a generic framework for parallel batch preprocessing of extracellular neuronal signals recorded by substrate microelectrode arrays
Source: Front Neuroinform. 2014 Mar 19;8:26. doi: 10.3389/fninf.2014.00026 (PMC3958706; doi:10.3389/fninf.2014.00026)
Supplement: Supplementary file 1 [file Presentation1.PDF]

# Supplemental Material

# for

# QSpike Tools: a Generic Framework for Parallel Batch Preprocessing of Extracellular Neuronal Signals Recorded by Substrate Microelectrode Arrays

*Mufti Mahmud*[1-2], *Rocco Pulizzi*[1], *Eleni Vasilaki*[1,4], *Michele Giugliano*[1,3-4,*]

[1]Theoretical Neurobiology and Neuroengineering Lab, Department of Biomedical Sciences, University of Antwerp, Universiteitsplein 1, B-2610 Wilrijk, Belgium;

[2] Institute of Information Technology, Jahangirnagar University, 1342 – Savar, Bangladesh

[3]Brain Mind Institute, Swiss Federal Institute of Technology Lausanne, CH-1015, Switzerland

[4]Department of Computer Science, University of Sheffield, S1 4DP Sheffield, UK.

[*] **Corresponding Author:** michele.giugliano@uantwerpen.be
Phone: +32 3 265 2616 Fax: +32 3 265 2669

# Summary of analysis for data file: 95_c_I5722_div20_sp_201307011931.mcd

This report has been automatically generated by a web-based analysis tool kit

Generated on Wednesday 4th December, 2013 at 15:17

## 1 Execution Log

Log file for the automated analysis of mcd file:
95_c_I5722_div20_sp_201307011931.mcd
==================================================
The analysis was initiated by: mufti
The analysis was launched from: mufti-vaio-wl.ua.ac.be.
During pre-processing Parallelization is set to: true
QUEUE used is: MEAs
The execution of the workflow started at: Wed Dec 4 15:05:09 CET 2013
At Wed Dec 4 15:05:09 CET 2013 the pre-processing process started
The execution of the pre-processing finished at: Wed Dec 4 15:11:16 CET 2013
Time taken for the pre-processing process is: 631 seconds
At Wed Dec 4 15:13:56 CET 2013 the post-processing and analysis processes started
Parallelization is set to: false
The execution of the post-processing and analysis finished at: Wed Dec 4 15:16:58 CET 2013
Time taken for the post-processing and analysis processes is: 182 seconds
Time taken to complete all the processes is: 973 seconds

## 2 Summary of the Activity

Activity Information Extracted from the Recording Data

---

Recording Duration : 1200100 ms
Number of Samples : 30002500
Sampling Rate : 25000 Hz
Number of Active Electrodes : 56
Number of Spikes : 105418
Number of Bursts : 165
Mean Burst Duration : 90.790303 ms
Standard Deviation of Burst Duration: 41.931074 ms
Mean Inter-Burst-Interval (IBI) : 7352.917317 ms
Standard Deviation of IBI : 2146.255105 ms
Burst Detection Threshold : 100

## 3 Results Summary

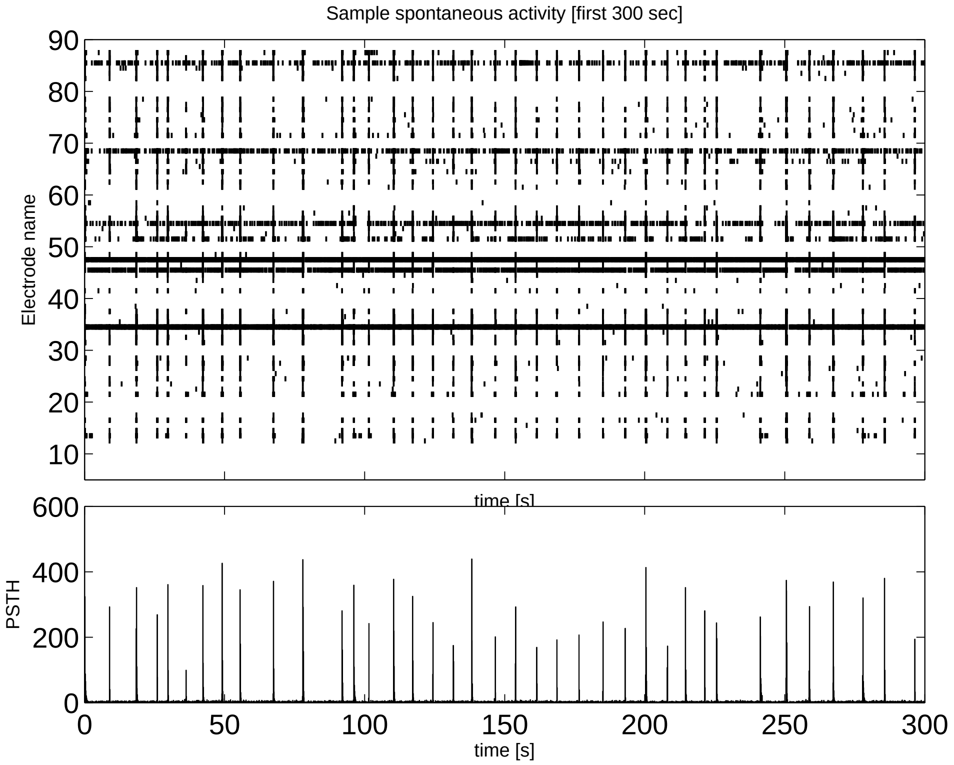


Figure 1: Sample activity (first 300 seconds) from the data file.

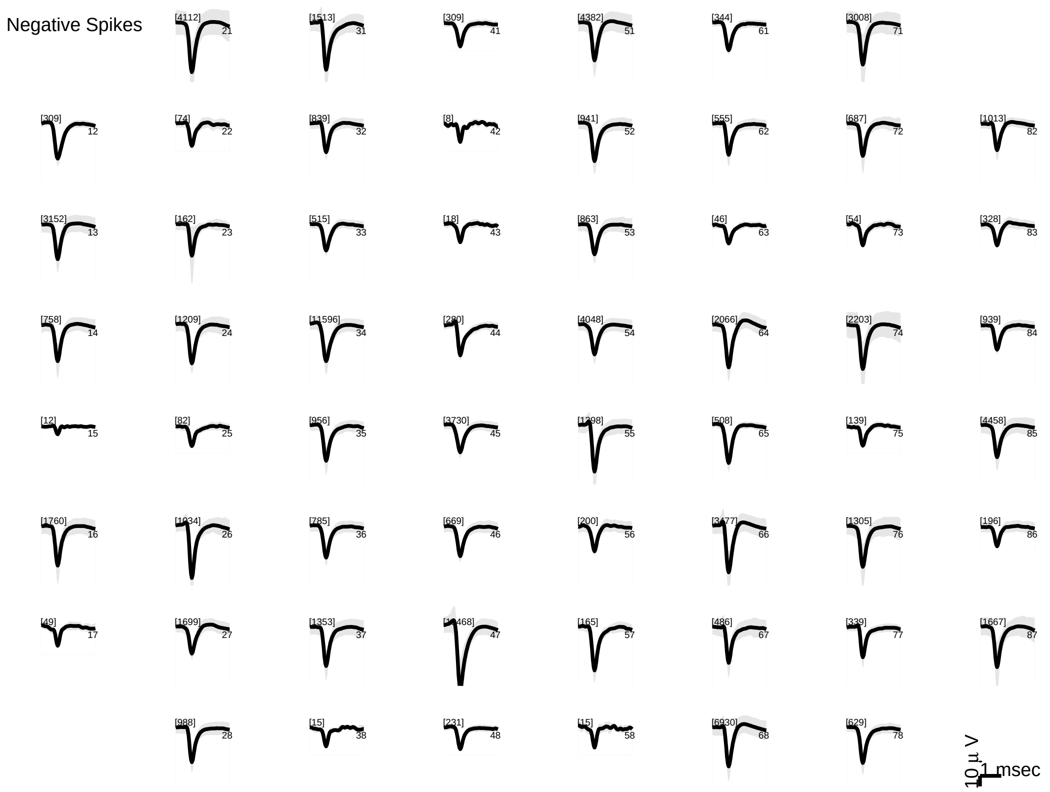


Figure 2: Negative spikes detected at distinct MEA microelectrodes.

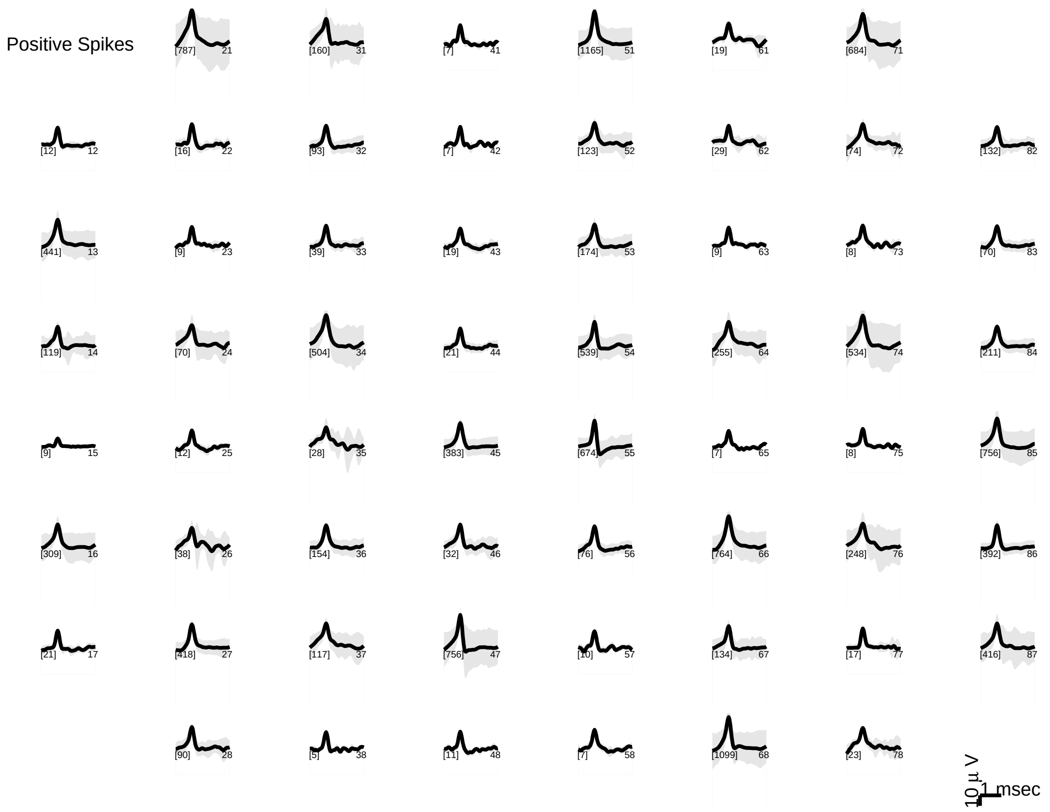


Figure 3: Positive spikes detected at distinct MEA microelectrodes.

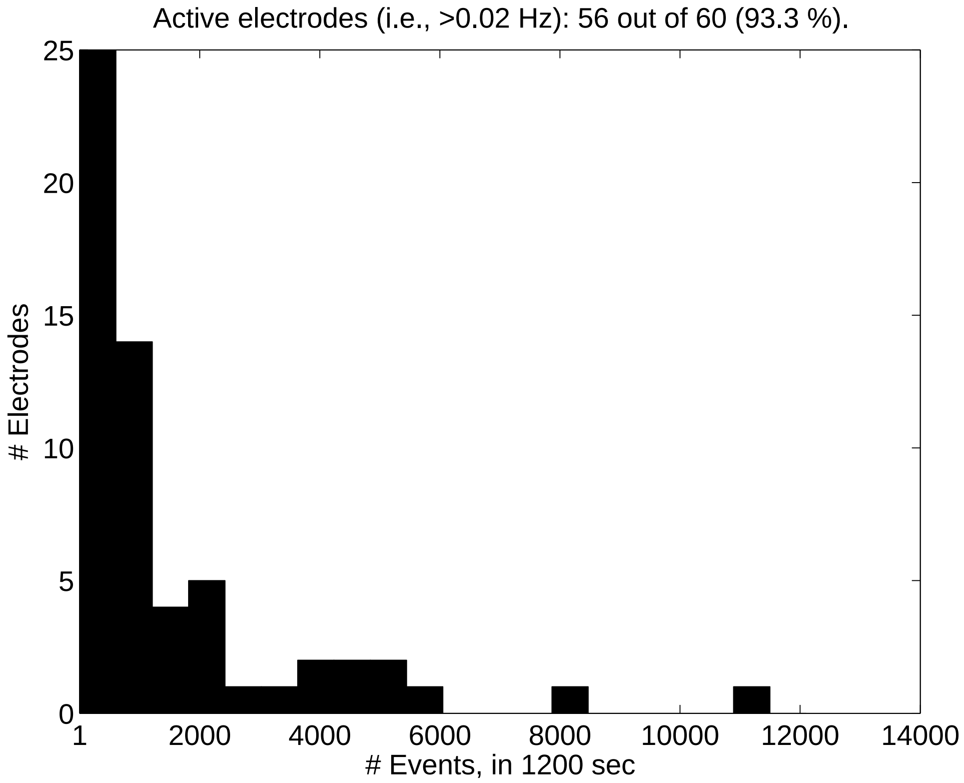


Figure 4: Histogram of the electrodes number where multi-unit activity was detected above 0.02 Hz.

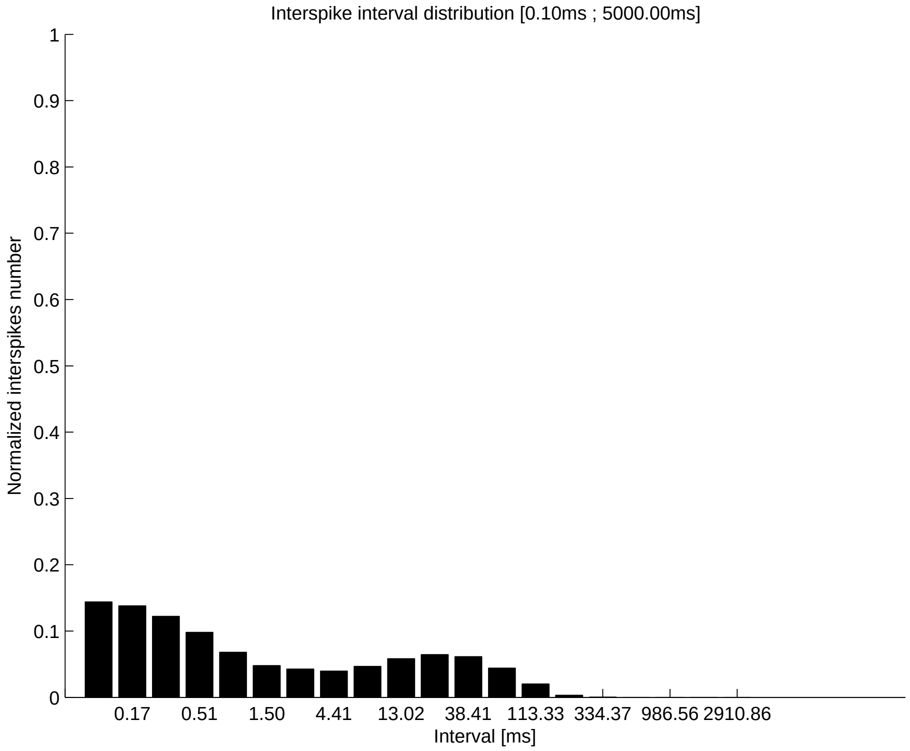


Figure 5: Inter spike interval (ISI) distribution of the detected events.

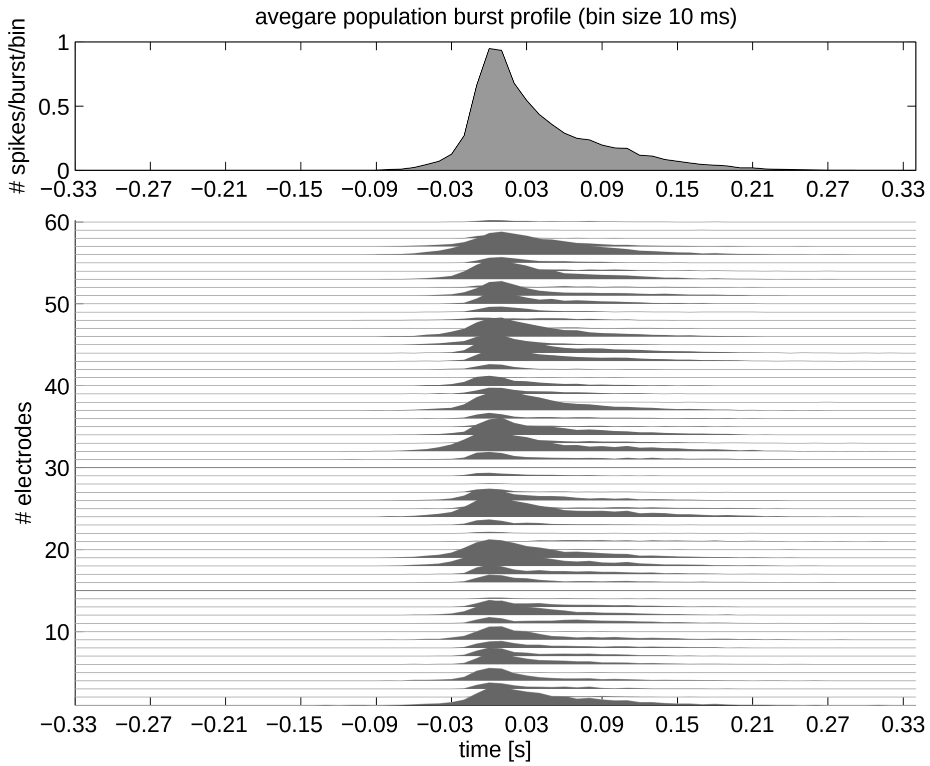


Figure 6: Burst trigerred instantaneous firing probability across electrodes.

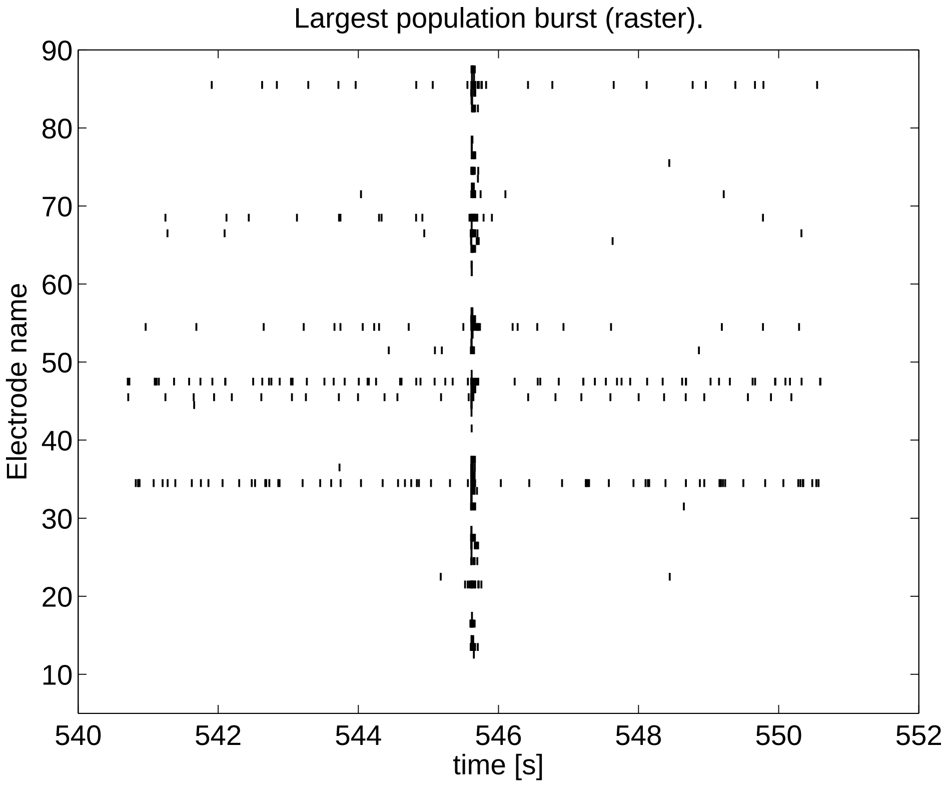


Figure 7: Multi-unit time raster diagram, centered around the most intense burst recorded.

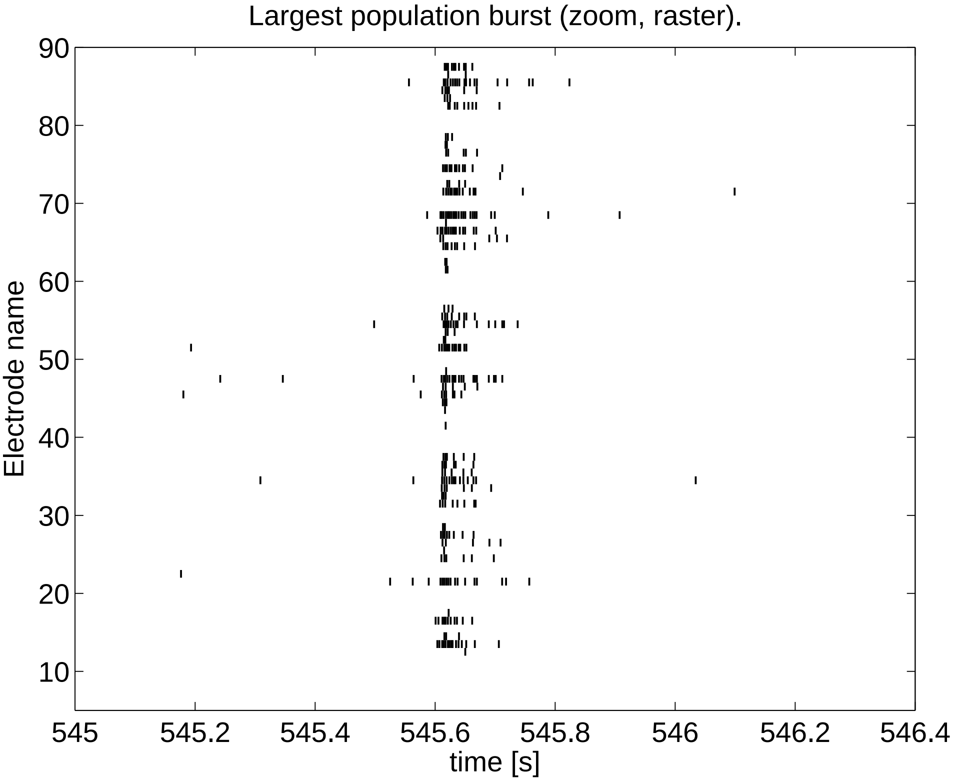


Figure 8: Detail of multi-unit time raster diagram, centered around the most intense burst recorded.

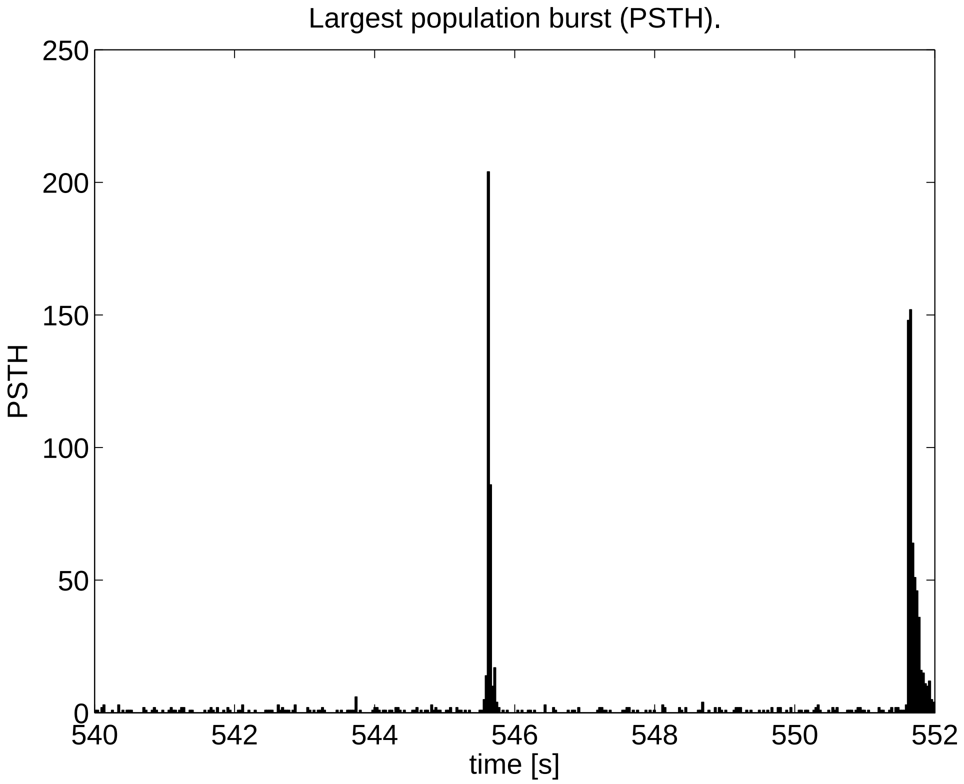


Figure 9: Multi-unit event time histogram, centered around the most intense burst recorded [bin size 3 ms].

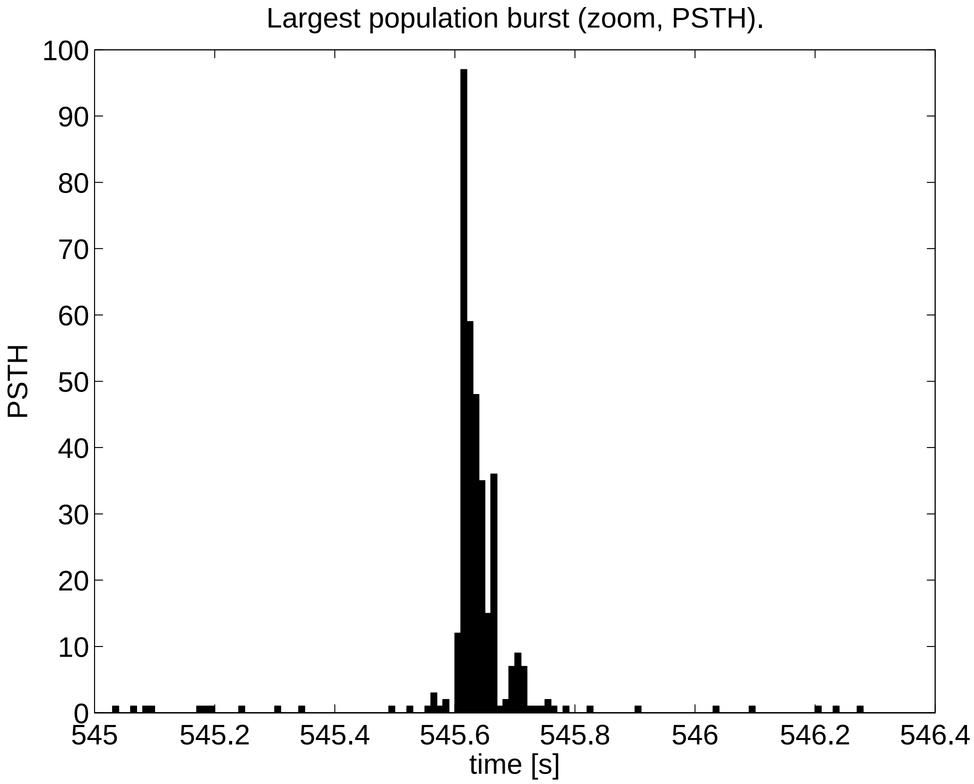


Figure 10: Detail of multi-unit event time histogram, centered around the most intense burst recorded [bin size 10 ms].

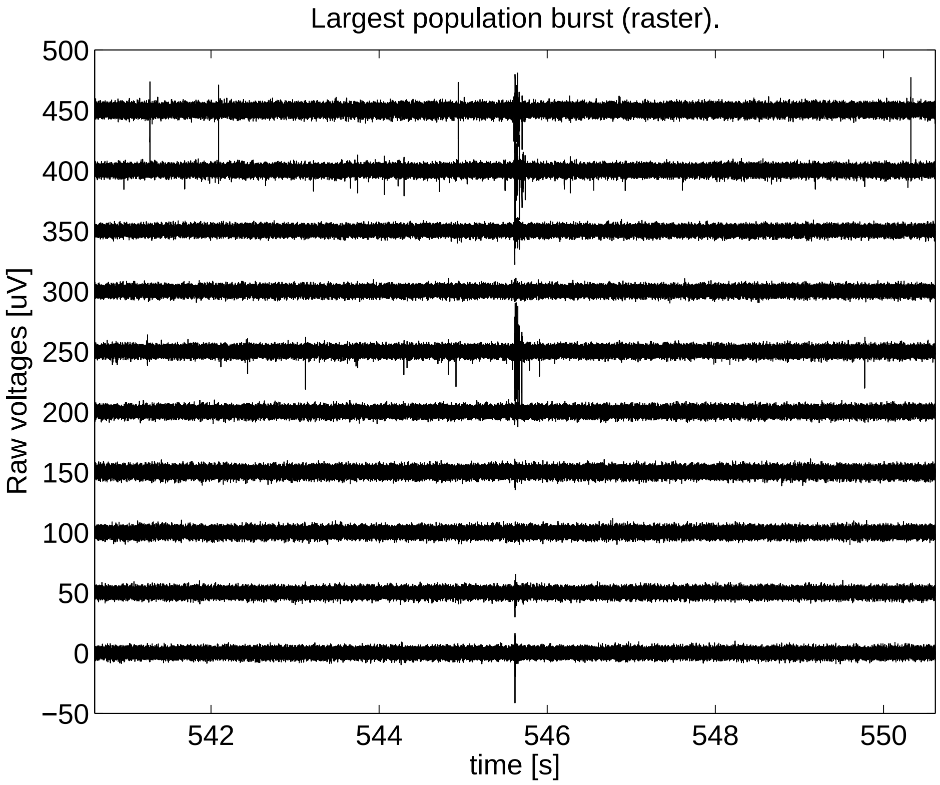


Figure 11: Corresponding raw voltage traces across MEA electrodes to the burst of Figures 7-10.

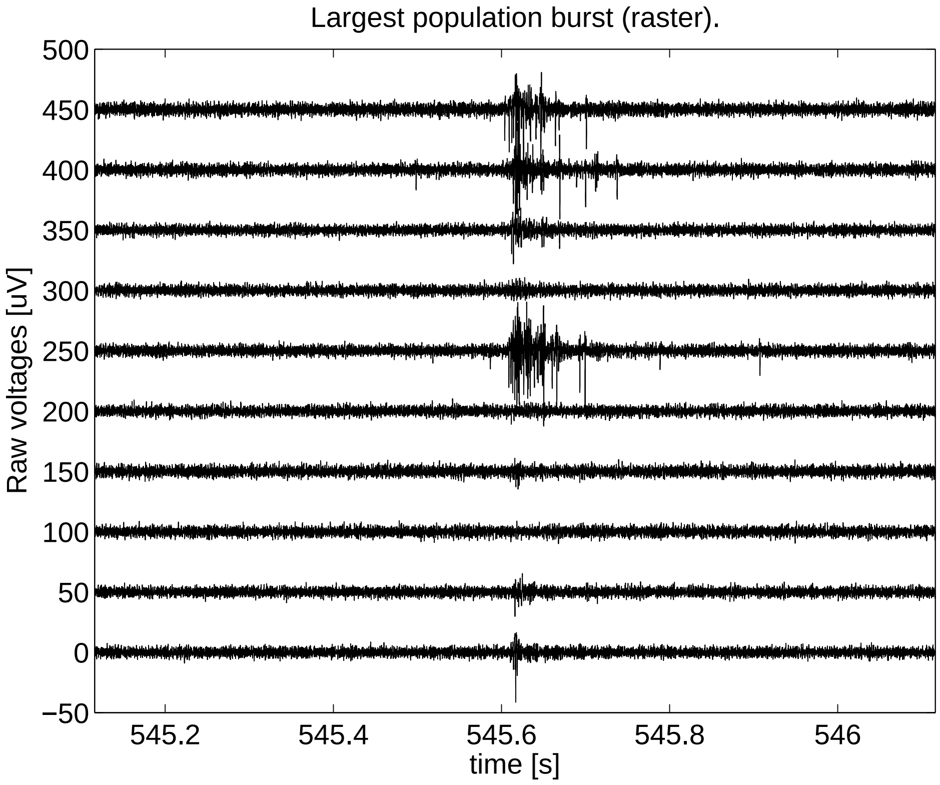


Figure 12: Detail of corresponding raw voltage traces across MEA electrodes to the burst of Figures 7-10.

# Installation Manual

# for

# QSpikeTool[1]

[1] As described in "QSpike Tools: a Generic Framework for Parallel Batch Preprocessing of Extracellular Neuronal Signals Recorded by Substrate Microelectrode Arrays" by *M. Mahmud, R. Pulizzi, E. Vasilaki, and M. Giugliano*

# Table of Contents

Introduction ........ 3
Install Ubuntu Server ........ 3
Install desktop Environment ........ 4
Install and configure the webserver ........ 4
  Install Apache 2 ........ 4
  Install Support for Scripting ........ 5
  Checking for proper installation ........ 6
  Configuration ........ 6
  Setting up basic authentication ........ 7
Install and configure the grid engine ........ 8
  Install Java Runtime Environment ........ 8
  Install Grid Engine ........ 8
Install and configure QSpikeTool ........ 13
  Install QSpikeTool ........ 13
  Install the supporting software ........ 14
    Install Matlab ........ 14
    Install LaTex ........ 16
  Installing libraries ........ 16
  Updating QSpikeTool with system specific information ........ 16
    Updating the index.html file ........ 16
    Updating the mcd_web_main.cgi file ........ 16
    Updating the transferInput.cgi file ........ 17

# Introduction

In the following sections you will be guided through the installation and configuration processes of the server ready to be used with QSpikeTool.

The different sections of this document will guide you to install and configure the various resources needed to run the QSpikeTool. You may jump to the required section to proceed with the installation process if you happen to have a configured system already. The sections are as follows:

- Install Ubuntu Server
- Install GUI for the Server
- Install and configure the webserver
- Install and configure the grid engine
- Install and configure QSpikeTool

# Install Ubuntu Server

First, you will need to download the current version of Ubuntu Server (http://www.ubuntu.com/start-download?distro=server&bits=64&release=latest). A 64 bit version is recommended. Once downloaded you need to create a bootable CD/DVD or USB flash.

Once done, put the disc in your drive, or insert the USB flash, and reboot your machine. Make sure that you set your BIOS to boot either from the CD/DVD or USB flash depending on which installation method you choose.

Once booted, just follow the steps shown in the images below:

On the first screen you can choose you language of install.

Select Install Ubuntu Server.

Select the language used for the installation process.

Select your geographical location.

Configure your keyboard.

The installation will detect and load any additional component.

The installation will detect the network hardware.

Type in the host name you want to configure your server as.

Type in the user's name.

Type in a password for the username.

Choose whether to encrypt your home directory or not. It is advisable not to encrypt home directory to facilitate data recovery in case of a crash.

Configure clock, and time zone.

During the detection process of the hardware and devices present in your system at certain point you will be presented with the disk(s) available: at this stage is where you partition your disk(s). Select Guided as it is the default method.

Select the partition of your choice where you would like to install the Ubuntu server.

The installation will copy data at this stage.

The installation will configure Apt Sources.List.

You can configure a Proxy. If you don't have a proxy, select Continue.

The installation will select the software being installed.

This is where you configure how to install updates. Recommended is the automatic method.

Select the software to be installed, OpenSSH and LAMP server is recommended.

Then the setup will perform Cleaning up and installation of Grub and Boot Loader.

Select continue in the Finishing step and remove the disk or USB.

Reboot your machine, and login with your login and password. You are now logged in to your new Ubuntu server!

## Install desktop Environment

If you have not selected to install the LAMP server during your server installation process, you can install a graphical interface using the following instructions:

First you need to make sure you have enabled Universe and multiverse repositories in /etc/apt/sources.list file. Once you have enabled you need to use the following command to install the GUI:

sudo apt-get update

sudo apt-get upgrade

sudo apt-get install ubuntu-desktop

The above command will install GNOME desktop.

## Install and configure the webserver

### Install Apache 2

Make sure your package repositories and installed programs are up to date by issuing the following commands:

apt-get update

apt-get upgrade --show-upgraded

Enter the following command to install the Apache 2 web server, its documentation and a collection of utilities.

apt-get install apache2 apache2-doc apache2-utils

Edit the main Apache configuration file to adjust the resource use settings.

File:/etc/apache2/apache2.conf

```
#
# KeepAlive: Whether or not to allow persistent connections (more than
# one request per connection). Set to "Off" to deactivate.
#
KeepAlive On

#
# MaxKeepAliveRequests: The maximum number of requests to allow
# during a persistent connection. Set to 0 to allow an unlimited amount.
# We recommend you leave this number high, for maximum performance.
#
MaxKeepAliveRequests 100

#
# KeepAliveTimeout: Number of seconds to wait for the next request from the
# same client on the same connection.
#
KeepAliveTimeout 15

##
## Server-Pool Size Regulation (MPM specific)
##

# prefork MPM
# StartServers: number of server processes to start
# MinSpareServers: minimum number of server processes which are kept spare
# MaxSpareServers: maximum number of server processes which are kept spare
# MaxClients: maximum number of server processes allowed to start
# MaxRequestsPerChild: maximum number of requests a server process serves
<IfModule mpm_prefork_module>
    StartServers          5
    MinSpareServers       5
    MaxSpareServers      10
    MaxClients          150
    MaxRequestsPerChild   0
</IfModule>

# worker MPM
# StartServers: initial number of server processes to start
# MaxClients: maximum number of simultaneous client connections
# MinSpareThreads: minimum number of worker threads which are kept spare
# MaxSpareThreads: maximum number of worker threads which are kept spare
```

## Install Support for Scripting

The following commands are optional, and should be run if you want to have support within Apache for server-side scripting in PHP, Ruby, Python, or Perl.

To install Ruby support, issue the following command:

apt-get install libapache2-mod-ruby

To install Perl support, issue the following command:

apt-get install libapache2-mod-perl2

To install Python support, issue the following command:

apt-get install libapache2-mod-python

If you need support for MySQL in Python, you will also need to install Python MySQL support:

apt-get install python-mysqldb

Your PHP application may require additional dependencies included in Ubuntu. To check for available PHP dependencies run "apt-cache search php", which will provide a list of package names and descriptions. To install, issue the following command:

apt-get install libapache2-mod-php5 php5 php-pear php5-xcache

Issue the following command to install the php5-suhosin package, which provides additional security to your PHP installation:

apt-get install php5-suhosin

If you're also hoping to run PHP with MySQL, then also install MySQL support:

apt-get install php5-mysql

## Checking for proper installation

Open a browser and type in localhost in your address bar and if the installation has been completed successfully, you will see a message showing that 'It Works!'.

## Configuration

Apache2 is configured by placing directives in plain text configuration files. These directives are separated between the following files and directories:

apache2.conf: the main Apache2 configuration file that contains global settings for Apache2.

conf.d: contains configuration files which apply globally to Apache2. Other packages that use Apache2 to serve content may add files, or symlinks, to this directory.

envvars: file where Apache2 environment variables are set.

httpd.conf: historically the main Apache2 configuration file, named after the httpd daemon. The file can be used for user specific configuration options that globally effect Apache2.

mods-available: this directory contains configuration files to both load modules and configure them. Not all modules will have specific configuration files, however.

mods-enabled: holds symlinks to the files in /etc/apache2/mods-available. When a module configuration file is symlinked it will be enabled the next time apache2 is restarted.

ports.conf: houses the directives that determine which TCP ports Apache2 is listening on.

sites-available: this directory has configuration files for Apache2 Virtual Hosts. Virtual Hosts allow Apache2 to be configured for multiple sites that have separate configurations.

sites-enabled: like mods-enabled, sites-enabled contains symlinks to the /etc/apache2/sites-available directory. Similarly when a configuration file in sites-available is symlinked, the site configured by it will be active once Apache2 is restarted.

In addition, other configuration files may be added using the Include directive, and wildcards can be used to include many configuration files. Any directive may be placed in any of these configuration files. Changes to the main configuration files are only recognized by Apache2 when it is started or restarted.

The server also reads a file containing mime document types; the filename is set by the TypesConfig directive, and is /etc/mime.types by default.

## Setting up basic authentication

You can set up a basic authentication service by setting up the .htaccess file. What is does is mainly password-Protect your directory with .htaccess.

Create a file called .htaccess in the directory you want to password-protect with the following content:

AuthUserFile /etc/apache2/.htpasswd
AuthGroupFile /dev/null
AuthName "Authentication Required to Login to the Web Analysis Toolkit"
AuthType Basic
require user username

```
AuthUserFile  /etc/apache2/.htpasswd
AuthGroupFile /dev/null
AuthName  "Authentication Required to Login to the Web Analysis Toolkit"
AuthType Basic
require user  username
/var/www/.htaccess (END)
```

NOTE: The username should be replaced with the desired name of the user.

Then create the file /etc/apache2/.htpasswd which contains the users that are allowed to login and their passwords. We do that with the htpasswd command:

htpasswd -c /etc/apache2/.htpasswd username

The -c flag is used only when you are creating a new file. After the first time, you will omit the -c flag, when you are adding new users to an already-existing password file. Otherwise you will overwrite the file!

The .htpasswd file will look like this:

```
username:$some encrypted text representing your password
/etc/apache2/.htpasswd (END)
```

Restart your webserver: sudo /etc/init.d/apache2 restart

# Install and configure the grid engine

## Install Java Runtime Environment

Before you install the grid engine, make sure that you have the appropriate version of the java runtime environment (jre) installed in your pc. If you are setting up your server using the information provided above, you will not have jre installed in your system and you need to manually do that. The easiest way is to install it with help of a package manager. You may select to install synaptic package manager for installing java. To install the synaptic package manager, use the command:

sudo apt-get install synaptic

Once the package manager is installed, open the Dashboard of Ubuntu and type 'Synaptic' and click the package manager icon shown. Type in java in the package manager and only select to install java jre (the jdk is not required).

## Install Grid Engine

Download grid engine from [http://gridscheduler.sourceforge.net](http://gridscheduler.sourceforge.net), at present it can be downloaded from: [http://dl.dropbox.com/u/47200624/respin/ge2011.11.tar.gz](http://dl.dropbox.com/u/47200624/respin/ge2011.11.tar.gz)

Extract the tarball which will give you a directory named 'ge2011.11'.

Place the extracted directory to /usr/local/ directory.

Change your working directory to /usr/local/ and create a symbolic link with the command: sudo ln –s ge2011.11 sge

View your '/etc/hosts' file. It should look like the following, and make sure that the ip address of your pc is provided before your host name (xxx.xxx.xxx.xxx in the picture).

```
127.0.0.1       localhost
xxx.xxx.xxx.xxx   ComputerName.domain  ComputerName

# The following lines are desirable for IPv6 capable hosts
::1     ip6-localhost ip6-loopback
fe00::0 ip6-localnet
ff00::0 ip6-mcastprefix
ff02::1 ip6-allnodes
ff02::2 ip6-allrouters
/etc/hosts (END)
```

Start the graphical user interface of the installer by typing the command: sudo /usr/local/sge/start_gui_installer

Follow the step-by-step guidelines below to set up your grid engine.

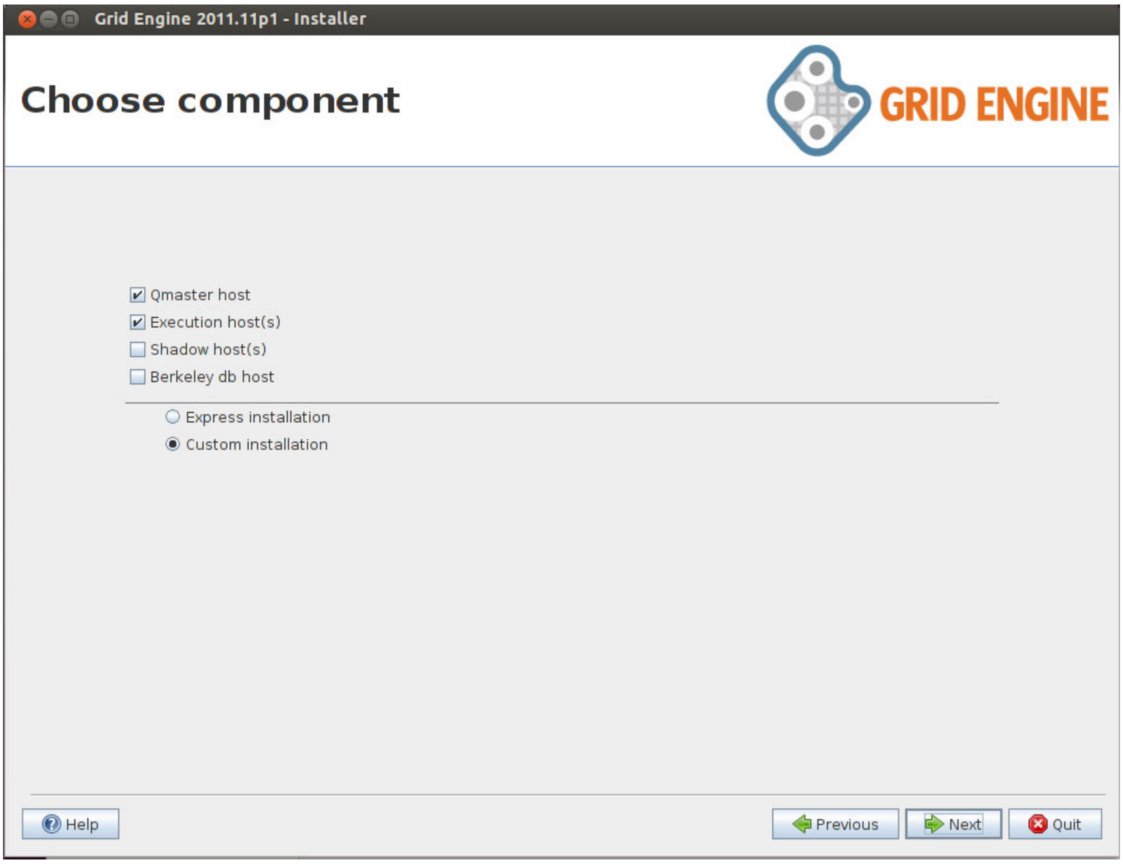


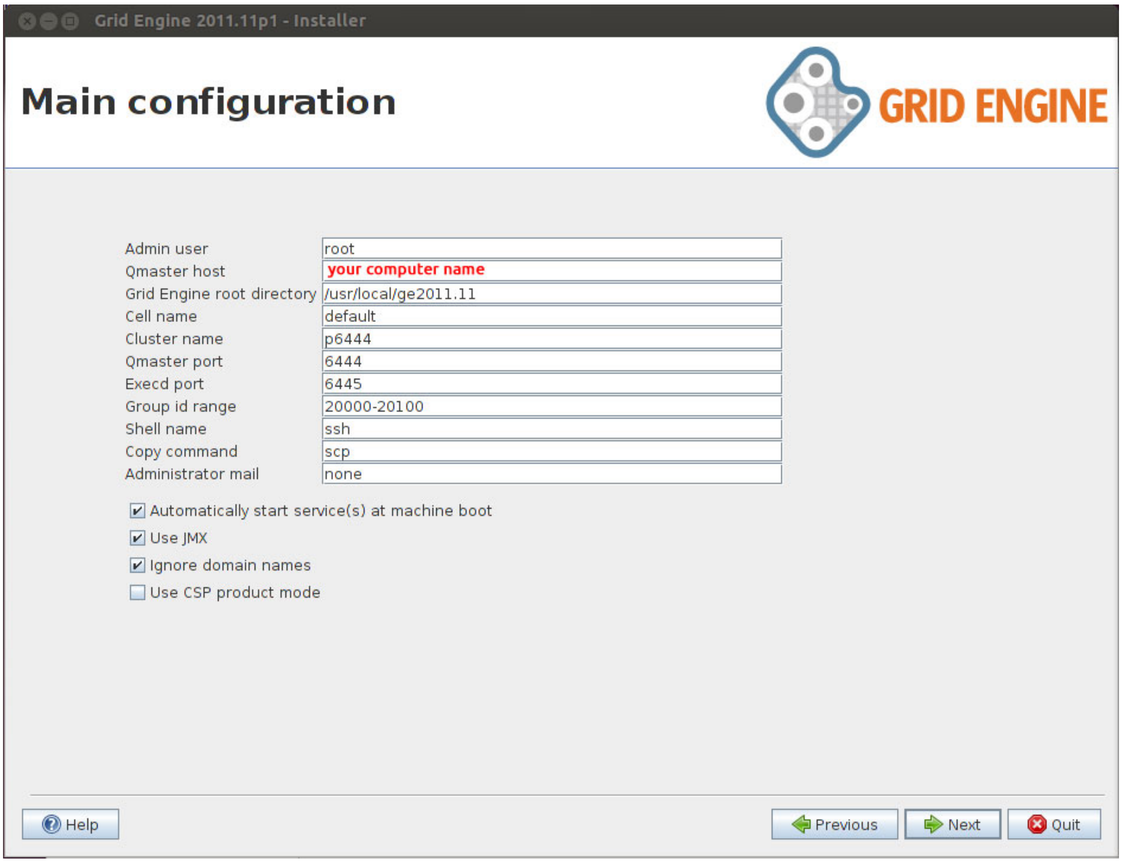

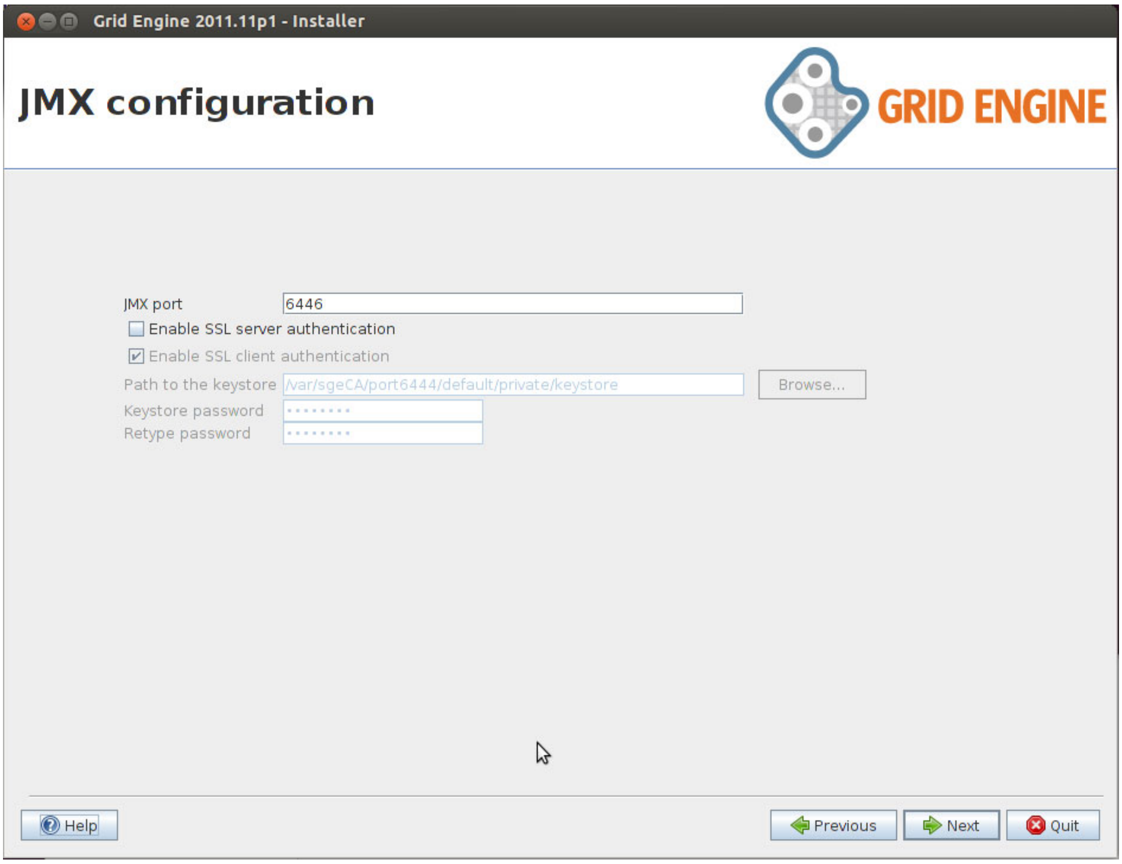


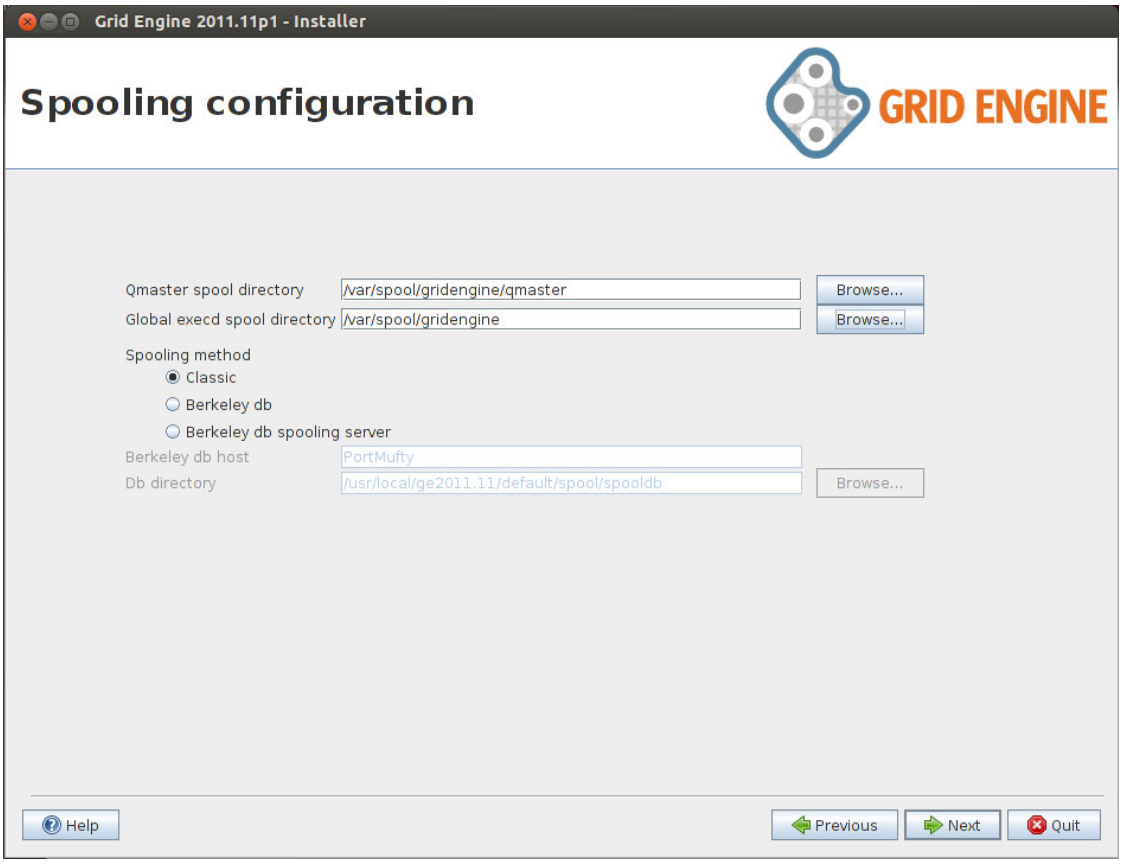


You can leave the default content of the SSL Certificate Configuration step as that feature has been disabled during the third step of the installation.

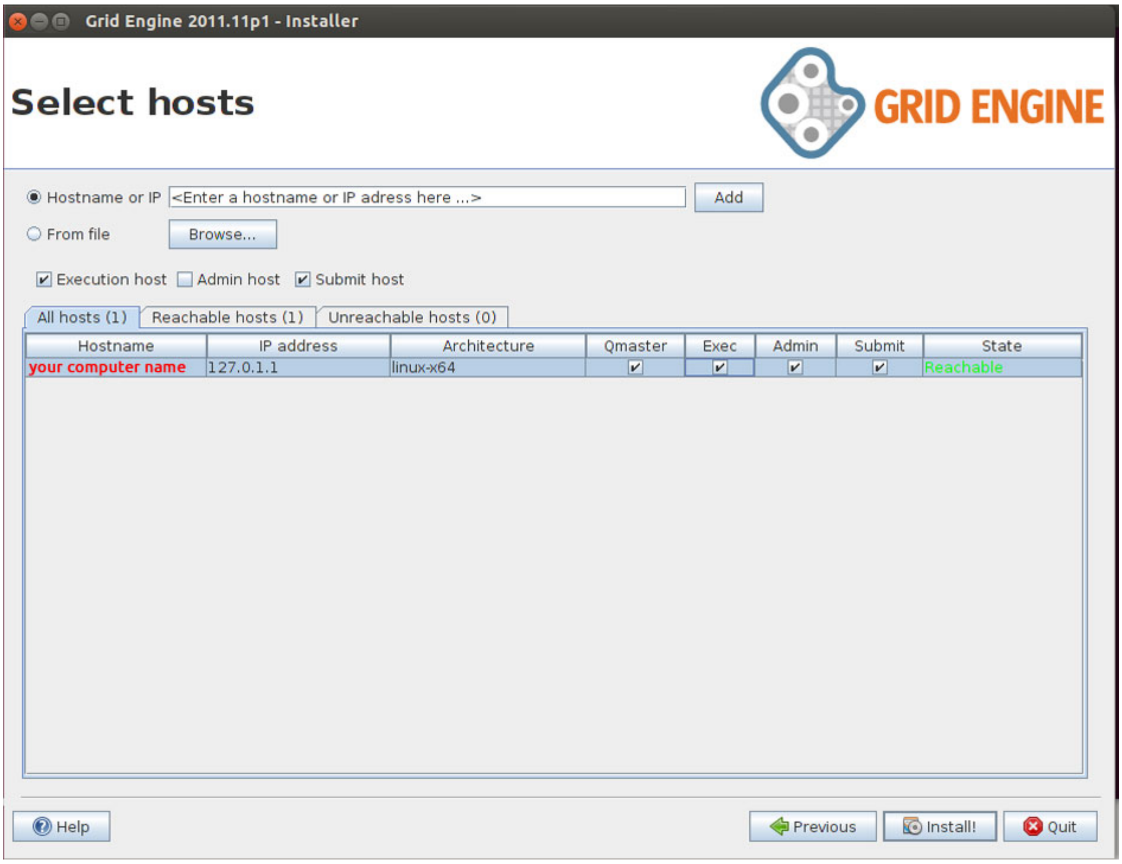


Click ‘Install’ and your installation will complete automatically.

Once completed save the final configuration file shown in html format, however, the same file also can be found at: /usr/local/sge/default/readme….html

This readme file will allow you to start and check the installation of the grid engine.

Edit the /etc/bash.bashrc file and add the following line at the end and it should look like the following picture.

source /usr/local/sge/default/common/settings.sh

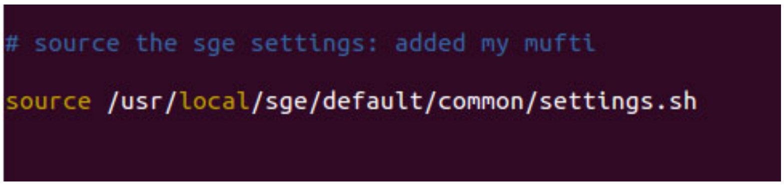


Before you start configuring your queues, install the required fonts to start qmon:

sudo apt-get install t1-xfree86-nonfree ttf-xfree86-nonfree ttf-xfree86-nonfree-syriac xfonts-75dpi xfonts-100dpi xfs xfstt libXft-dev libXext-dev python python-dev gcc g++ make binutils libx11-dev libxpm-dev libxft-dev libxext-dev build-essential

Start the graphical queue monitor using the command ‘qmon’ to configure queues.

Create the queues by carefully following instructions shown in the pictures:

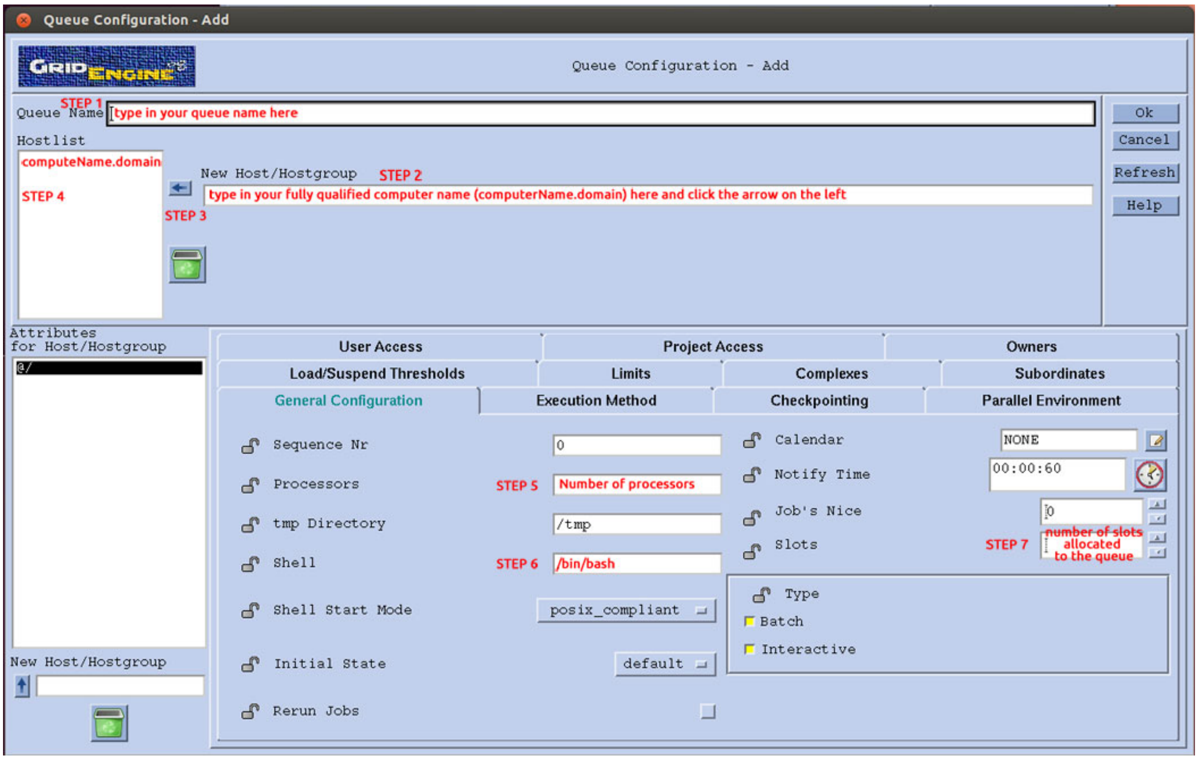


NOTE: Step 7 value should be less than or equal to the value provided in Step 5.

Click 'Ok' to create the queue with the specified name. You may create as many queues as you desire. Remember to use the number of slots in and queue name while personalizing your web-interface.

After setting up the queues, the users should be configured. Click the 'User' button in QMON and add the 'www-data' user in the 'Userset' and 'User' as shown in the following pictures:

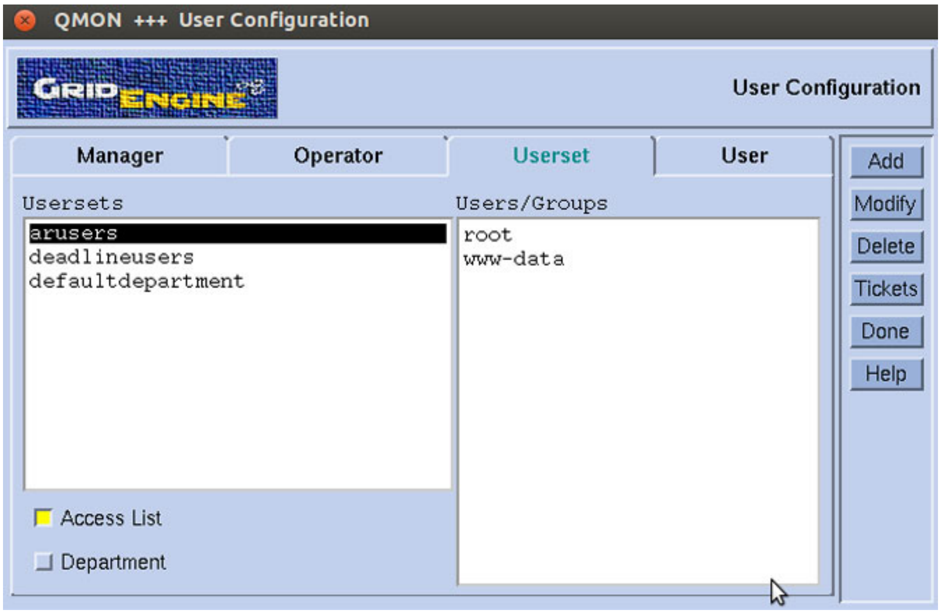

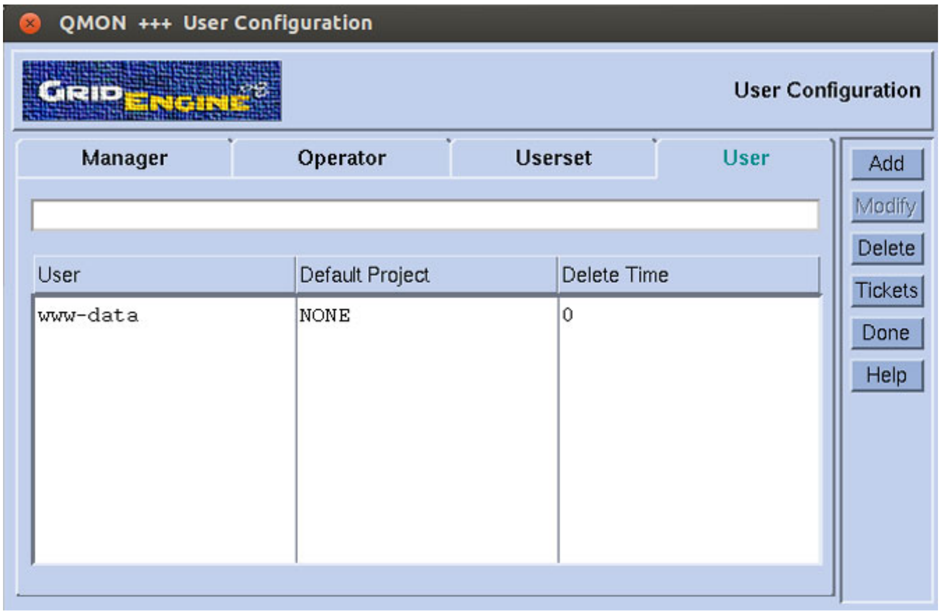


Now you have set up your queue system to run the QSpikeTool.

# Install and configure QSpikeTool

## Install QSpikeTool

To install and configure the QSpikeTool, you need to follow the following instructions:

Create directories with the commands (your current working directory should be /):

mkdir /data1
mkdir /data1/WWWINTERFACE
mkdir /data1/WWWINTERFACE/INPUT_MCD_FILES
mkdir /data1/WWWINTERFACE/OUTPUT_PREPROCESSED_FILES

Extract the downloaded zipped file qspiketool.rar.

Copy the required 'QSpikeTool' directory to '/data1/WWWINTERFACE/'.

Copy the html files residing in the 'www' directory to /var/www directory.

Copy the script files residing in the 'cgi-bin' directory to /usr/lib/cgi-bin directory.

Change the ownership of the created directories for data accessing and writing by 'www-data' user:

- add user 'www-data' to 'plugdev' group: sudo usermod –a –G plugdev www-data
- change ownership of the directories: sudo chown –R root:plugdev /data1/
- change ownership of the QSpikeTool directory: sudo chown www-data:plugdev /data1/WWWINTERFACE/QSpikeTool

Change the privileges of the directories as follows:

- sudo chmod 775 /data1

- sudo chmod –R 777 /data1/WWWINTERFACE/
- sudo chmod 755 /data1/WWWINTERFACE/QSpikeTool

Before running the codes, make sure that the files have proper execution rights, if not you may change the execution rights of files in a directory using the command 'chmod 755 *'.

For noninteractive secure data transfer install 'sshpass' by the command: sudo apt-get install sshpass.

You may now try to check if the website is set properly by opening a browser and typing in the ip address of the server you have just setup: you should see the welcome page.

Before you run the codes, you need to make sure that your network domain is set properly and the computers' domains are listed in the '/etc/resolv.conf' file, if not, set the search domain using the following command:

sudo vim /etc/resolv.conf

And add the domain name(s) of the acquisition and/or user pcs in the 'search' command as shown in the picture:

```
# Dynamic resolv.conf(5) file for glibc resolver(3) generated by resolvconf(8)
#     DO NOT EDIT THIS FILE BY HAND -- YOUR CHANGES WILL BE OVERWRITTEN
nameserver xxx.xxx.xxx.xxx
nameserver xxx.xxx.xxx.xxx
search domain1 domain2 domain3
/etc/resolv.conf (END)
```

## Install the supporting software

To run the QSpikeTool toolkit, you need to have Matlab (www.mathworks.com) and latex installed in your system. If you don't have them installed, follow the following instructions to install them:

### Install Matlab

To install Matlab for Linux x64 bit computer, first you would need to download it from the official website of Matlab; the latest release is R2013b and can be downloaded from http://www.mathworks.com/downloads/web_downloads/get_release?release=R2013b

After entering the required login information in the link provided above, follow the instructions given below to download and install Matlab:

Step 1: Create a new directory, such as ~/mathworks_downloads, to store the downloaded installation files (where ~ is the home directory):

mkdir ~/mathworks_downloads
cd ~/mathworks_downloads

Step 2: Click "Download" button for each file listed in the website and choose to save into the download directory created in Step 1. Download all listed products, including all _common.zip and _glnxa64.zip files, into the same directory.

Step 3: Execute the following command to extract the installer:

unzip matlab_R2013b_glnxa64_installer.zip

Step 4: Execute the following command to begin the graphical installer:

./install

NOTE: You must run the installation as root and install MATLAB in the /usr/local directory.

Step 5: You want to install symbolic links to the MATLAB executables into the system path, such as in /usr/local/bin.

Step 6: You need to install a Standalone Named User license and do not run the activation client as root user. After installation, choose to exit without activating. The end user will be prompted to activate when launching MATLAB for the first time.

Step 7: Add a symbolic link to your /usr/local directory to avoid version problem using the command: sudo ls –s /usr/local/MATLAB/<R201..> /usr/local/matlab

In the above command the <R201..> should be replaced with the exact release of matlab installed in your system.

Step 8: Make sure that your Matlab installation is executable from the Linux command line. If it doesn't start and shows you an error saying java exception and can't write the preference, create a '.matlab' directory in your home directory and check for proper ownership of the directory using the following commands:

mkdir ~/.matlab
chown –R <user>:<user> ~/.matlab

where <user> is the user name.

Step 9: Try running Matlab as the 'www-data' user using the following command in your command line:

sudo –u www-data /usr/local/matlab/bin/matlab

If you encounter an java exception and write protection error of preference as stated in Step 8, do the following:

Create a directory named '.matlab' in the /var/www directory using the command: sudo mkdir /var/www/.matlab

Change the ownership of the directory to the www-data user using the command: sudo chown –R www-data:www-data /var/www/.matlab

Try to run Matlab again as www-data user using the command: sudo –u www-data /usr/local/matlab/bin/matlab and this should start the Matlab GUI as www-data user.

Step 10: In the Matlab GUI, add the directories '/data1/WWWINTERFACE/QSpikeTool' and '/data1/WWWINTERFACE/QSpikeTool/matlab' in your path.

### Install LaTex

Follow the instructions given below to install LaTex in your system.

Install latex from the command line using the command: sudo apt-get install latexmk

Install recommended fonts using command: sudo apt-get install texlive-fonts-recommended

Install texlive extra to work with some required packages using command: sudo apt-get install texlive-latex-extra

## Installing libraries

The library files from boost1.42.0 C++ library are required to run the QSpikeTool smoothly. The required files are placed in the extracted directory as a subdirectory named ‘boostlib’. Copy the library files present in the boostlib directory and paste them in /usr/lib directory.

## Updating QSpikeTool with system specific information

### Updating the index.html file

Modify the ‘index.html’ file located in the /var/www/ directory and it should look like this:

```
<tr>
        <td>
                <p align="left" >
                        <label for="username">Username: </label>
                        <select name="username" id="user_list">
                        <option value="select">Select from here</option>
                        <option value="username">user full name</option>
                        </select>
                </p>
            <br>
                <p align="left">
                        <input name="user_name" type="submit" value="Proceed to the analysis page">
                </p>
        </td>
</tr>
```

Change the ‘username’ and ‘user full name’ with the username and the full name of the users to whom you would like to provide access to your system.

### Updating the mcd_web_main.cgi file

#### *Adding users*

According to how you have updated your index.html file, you need to modify the corresponding ‘mcd_web_main.cgi’ file located in the /usr/lib/cgi-bin/ directory. Type in the usernames and user full names you have entered in your index.html file.

NOTE: Before a user can be added to the index.html or mcd_web_main.cgi files, the user must have a user account in the cluster, i.e., a user account must be created for the user in the cluster to allow the user the access to the web based analysis.

```
if [ "$regUser" == "$username" ] ; then
  if [ "$username" == "username" ] ;  then
    usern="user full name"
  elif [ "$username" == "username2" ] ; then
    usern="user full name 2"
  fi
fi
```

### *Specifying queues to use*

You also need to provide the queue information on this file based on the queues that you have created earlier. Find the following code segment in the mcd_web_main.cgi file and edit according to your specifications of queues.

```
echo '<input name="queue" type="radio" value="queue name">'
echo 'display queue name<br>'
```

NOTE: The queue name should be exactly the same as specified in the grid engine. Mismatch will cause in errors and your analysis toolkit will not run!

### Updating the transferInput.cgi file

The transferInput.cgi file located at /usr/lib/cgi-bin/ provides the interface to transfer data files from the acquisition computer to the cluster. The acquisition PCs must be specified before a user can use them to transfer the files. Edit the file and enter the appropriate information to access the computers.

```
'<input name="acqPC" type="radio" value="registered-pc-name1" checked>name to display 1</br>'\
'<input name="acqPC" type="radio" value=" registered-pc-name2"> name to display 2'\
```

If you are not sure about the name of the acquisition PC, you can find that using the command: nslookup xxx.xxx.xxx.xxx |grep "name"

This should provide the registered name of the computer in the network. Use only the name without the domain in the registered-pc-name.
